# Creatine kinase B, a downstream effector of c-Myb, controls migration of osteosarcoma cells via regulation of N-cadherin

**DOI:** 10.1186/s12935-025-04087-0

**Published:** 2025-12-05

**Authors:** Jana Pokludová, Petr Lapčík, Iva Staniczková Zambo, Jiří Kohoutek, Danica Zapletalová, Peter Múdry, Dagmar Adámková, Jakub Červinka, Tomáš Loja, Matej Lexa, Jan Verner, Jan Šmarda, Pavel Bouchal, Lucia Knopfová, Petr Beneš

**Affiliations:** 1 Department of Experimental Biology, Faculty of Science, Masaryk University, Brno, Czech Republic; 2 International Clinical Research Center, St. Annes University Hospital, Brno, Czech Republic; 3 Department of Biochemistry, Faculty of Science, Masaryk University, Brno, Czech Republic; 4 1st Department of Pathology, St. Anne´s University Hospital and Faculty of Medicine, Masaryk University, Brno, Czech Republic; 5 Department of Pediatric Oncology, University Hospital Brno and Faculty of Medicine, Masaryk University, Brno, Czech Republic; 6 Masaryk Memorial Cancer Institute and Faculty of Medicine, Masaryk University, Brno, Czech Republic; 7 CEITEC – Central European Institute of Technology, Masaryk University, Brno, Czech Republic; 8 Faculty of Informatics, Masaryk University, Brno, Czech Republic

## Abstract

**Background:**

We have recently identified transcription factor c-Myb as a negative prognostic factor in osteosarcoma (OSA) patients associated with metastatic disease. Transcriptomic analysis identified creatine kinase B (*CKB*) as one of the most deregulated genes in OSA cell lines with depleted *MYB*. CKB is a component of the creatine/phosphocreatine system that plays a key role in maintaining cellular energy homeostasis and energy transport to sites with high demand. This study was therefore conducted to investigate the functional significance of CKB in OSA.

**Methods:**

Deregulation of *CKB* by c-Myb in OSA cells was analyzed using gain-of-function/loss-of-function approach. Transactivation of the *CKB* promoter by c-Myb was assessed using a reporter assay. CRISPR/Cas9, RNAi and cyclocreatine were used to inhibit the expression/activity of CKB in OSA cells. Cell growth, colony-forming capacity, cell migration, chemosensitivity in vitro and metastatic capacity in vivo was examined. CKB protein effectors were identified using liquid chromatography-mass spectrometry (LC-MS) in data-independent acquisition-parallel accumulation serial fragmentation mode.

**Results:**

*CKB* was validated as c-Myb target in OSA cell lines. Depletion of *CKB* using CRISPR/Cas9 resulted in slower migration of OSA cells in vitro and reduced metastatic capacity in immunodeficient mice. siRNA and cyclocreatine inhibited OSA cell migration as well but in this case, cell proliferation was also reduced. A total of 8474 protein groups were quantified, with 147 downregulated and 143 upregulated protein groups associated with the *CKB* knockout phenotype. The deregulated proteins were enriched for those associated with cell migration and motility. N-cadherin, an established regulator of cell migration, was identified as a target of CKB signaling and its role in OSA cell migration and metastasis was confirmed.

**Conclusion:**

c-Myb – CKB – N-cadherin axis was identified as pathway regulating OSA cell migration and metastasis.

**Supplementary Information:**

The online version contains supplementary material available at 10.1186/s12935-025-04087-0.

## Introduction

Osteosarcoma (OSA) is the most common primary bone tumor that mainly affects two distinct patient groups, young adolescents and people over 50 years of age. The 5-year survival rate has reached a plateau in patients with localized disease ranging from 60 to 70% since the introduction of systematic chemotherapy [1]. Unfortunately, OSA is a highly aggressive tumor, and 15–20% of newly diagnosed patients already present with metastasis, mostly in the lungs [2]. The 5-year overall survival rate of patients with metastatic disease is below 25%, largely unchanged over the past 20 years [3]. The stagnation of current treatment options over recent decades is namely attributed to the heterogeneity and complexity of OSA somatic genome with only few common targetable alterations identified. Furthermore, it is also due to an incomplete understanding of the factors that drive the metastatic progression [1, 4].

In a recent study, we identified c-Myb, a transcription factor and an oncoprotein encoded by *MYB* gene, as a negative prognostic factor in OSA patients associated with metastatic disease. *MYB*/c-Myb was upregulated in metastatic OSA cell lines compared to their non-metastatic counterparts, and its knockout resulted in altered proliferation, migration, and chemosensitivity. To investigate the mechanism of c-Myb action and to identify potentially druggable effectors, we performed RNAseq analysis of OSA control and *MYB* knockout 143B and SAOS-LM5 cells and identified a set of c-Myb-regulated genes. Downregulation of creatine kinase B (CKB) was among the top hits in both cell lines [5].

CKB is a widely expressed cytosolic isoform of creatine kinase (CK), an enzyme that catalyzes a reversible transfer of the high-energy phosphate group between ATP and creatine. The creatine/creatine kinase/phosphocreatine system plays a key role in cellular energy buffering, energy homeostasis and energy transport to sites of high demand [6]. Aberrant expression of CKB has been observed in many cancers, where it has been associated with the regulation of cancer cell metabolism, proliferation, survival, plasticity, and motility [6–8]. Although a link between CKB and cancer diseases was proposed many years ago, the underlying molecular mechanism has only been uncovered in the last decade and may differ between cancer types [7–11].

In this study, we investigated the c-Myb – CKB axis in OSA and confirmed that CKB expression is regulated by the c-Myb oncoprotein in OSA cell lines and that both genetic and pharmacological inhibition of CKB reduces cell migration by decreasing N-cadherin, a mesenchymal marker associated with poor prognosis and metastasis in this aggressive bone tumor.

## Materials and methods

### Cell culture and chemicals

The OSA cell lines SAOS-2, MG63, and Dunn along with their metastatic variants SAOS-LM5, SAOS-LM5 lacZ mCherry, MG63-M8 and Dunn-LM8 were generously provided by Bruno Fuchs. SAOS-LM5 were employed to assess *CKB*/CKB expression by qPCR and Western blotting. In other experiments, the SAOS-LM5 lacZ mCherry cell line was used. All cell lines were maintained at 37 °C with 5% CO_2_ in DMEM medium (Sigma-Aldrich, St. Louis, MO) with 10% fetal bovine serum (FBS) (Invitrogen, Carlsbad, CA), 2 mM l-glutamine, 100 U/ml penicillin and 100 µg/ml streptomycin (Lonza, Basel, Switzerland). Cell line authentication was performed by Generi Biotech using short tandem repeat (STR) profiling. Cyclocreatine (CCr; 2-imino-1-imidazolidine acetic acid) (Sigma-Aldrich) was used at a final concentration of 10 mM.

### Derivation of *CKB* knockout cells

To establish *CKB* KO cells, quide RNA (gRNA) for CRISPR/Cas9 editing was designed using the CRISPOR web-based platform [12]. Complementary 25 bp forward and reverse oligonucleotides (“CRISPR *CKB*”, Supplementary material 1) were cloned into the *Bbs*I restriction site of the pSpCas9(BB)-2A-GFP (PX458) plasmid (Addgene) [13]. Oligonucleotides targeting GFP (”CRISPR scrambled”, Supplementary material 1) were used to construct a control plasmid (Supplementary material 2) [14]. SAOS-LM5 cells were transfected using Lipofectamine™ LTX Reagent (Invitrogen). Two days post-transfection, GFP-positive cells were sorted into a 24-well plate using the FACSAria Fusion cell sorter (BD Biosciences, Franklin Lake, NJ), expanded and cloned by a limiting dilution. CKB depletion in individual clones was verified by Western blot analysis. Genomic DNA was extracted from the *CKB* KO cells and small insertions/deletions within the *CKB* target sequence were validated by Sanger sequencing.

### Derivation of cells with deregulated expression of *MYB*

143B and SAOS-LM5 *MYB* KO cells were described previously [5]. The pcDNA4/TO-h*MYB* plasmid (Supplementary material 2) was obtained by cloning the human *MYB* coding sequence from pcDNA3-h*MYB* [14] using *Kpn*I/*Xba*I into pcDNA4/TO (Invitrogen). SAOS-2 *MYB*up cells were transfected with pcDNA4/TO-h*MYB* and selected with 500 µg/ml Zeocin for 14 days. Single cell clones were obtained by limiting dilution, and overexpression of *MYB*/c-Myb was confirmed by qPCR and Western blot analysis.

### siRNA-mediated silencing

Lipofectamine™ RNAiMAX Transfection Reagent (Invitrogen) was used for siRNA transfection with the following oligonucleotides to target *CKB* (10 μM, s3084, Invitrogen), *CDH2* (10 μM, s2771, Invitrogen) or control siRNA (10 μM, *Silencer*™ Select Negative Control No. 1 siRNA, Invitrogen). The cells were transfected according to the manufacturer’s instructions and the siRNA-silenced cells were analyzed 48 h later.

### Western blotting

Cells were lysed and Western blot analysis was performed as described previously [15]. The following primary and secondary antibodies were used: CKB (ab92452, Abcam, Cambridge, UK), N-cadherin (ab76011, Abcam), p21 (CST 2947, Cell Signaling Technology, Danvers, MA), p53 (DO-1 clone, Masaryk Memorial Cancer Institute) [16], α-tubulin (T9026, Sigma-Aldrich), horseradish peroxidase-conjugated mouse (A9044, Sigma-Aldrich) or rabbit secondary antibodies (A6154, Sigma-Aldrich). The Clarity™ Western ECL Substrate (Bio-Rad, Hercules, CA) was used to visualize the signal.

### RNA isolation and quantitative PCR (qPCR)

Total RNA was extracted from collected cells using the GenElute™ Mammalian Total RNA Miniprep Kit (Sigma-Aldrich). cDNA was synthesized using the QuantiTect^®^ Reverse Transcription Kit (Qiagen, Redwood City, CA). qPCR was carried out as described previously [5] with primers spanning exon-exon junctions (Supplementary material 3). h*GAPDH/*m*Gapdh* was used as an internal control. Relative gene expression levels were calculated using the 2^−ΔΔCt^ method.

### Transactivation luciferase assay

A 913 bp fragment of the human *CKB* promoter (PCR primer sequences listed in Supplementary material 1) was inserted into the *Kpn*I/*Sac*I restriction sites of the pGL3-basic vector (Promega, Madison, WI) to generate the luciferase reporter plasmid pGL3-h*CKB*promoter (Supplementary material 2). SAOS-LM5 cells were co-transfected with pGL3-h*CKB*promoter and pcDNA3-h*MYB* or pcDNA3 (control plasmid) using Lipofectamine^®^ LTX (Invitrogen). Luciferase activity was measured 24 h post-transfection and normalized to total protein content, determined by the DC protein assay (Bio-Rad), as previously described [14].

### Cell proliferation

2 × 10^5^ SAOS-LM5 cells and their *CKB* KO derivatives were cultured in 6-well plates for 96 h. The cells were counted every 24 h using the CASY^®^ cell counter (Roche).

### Chemosensitivity of cells

2 × 10^5^ SAOS-LM5 cells and their *CKB* KO variants were seeded into 6-well plates and treated with LD_50_ of cisplatin (CDDP; 20 µM; Sigma-Aldrich), doxorubicin (DOX; 400 nM; Sigma-Aldrich) or methotrexate (MTX; 100 nM; Sigma-Aldrich) for 3 days (CDDP and DOX) or 4 days (MTX). Cells were collected and stained with 50 nM SYTOX™ Green (Thermo Fisher Scientific, Waltham, MA) for 10 min. Cell viability was assessed by flow cytometry (BD FACSVerse™, BD Biosciences).

### Colony-forming assay

1 × 10^3^ SAOS-LM5 cells and their *CKB* KO variants were seeded in 5 ml Petri dishes and cultured for 14 days. Medium was refreshed three times per week. Colonies were fixed and stained using 0.05% crystal violet in formaldehyde and counted.

### Wound-healing migration assay

SAOS-LM5 or MG63-M8 cells were seeded into 24-well plates and cultured for 24 h to form confluent monolayers. Mechanical scratches were performed using 1000 µl pipette tips. Images were captured every 3 h over a 12-hour period, and wound closure was quantified using ImageJ software (U. S. National Institutes of Health, Bethesda, Maryland, USA). For experiments involving CCr, cells were pre-incubated in CCr-containing medium for 24 h prior to the scratch assay, and CCr remained present in the medium throughout the assay. In the case of siRNA experiments, transfection was performed 48 h before initiating the scratch assay.

### xCELLigence migration assay

Real-time monitoring of cell migration was performed using RTCA CIM plates (Agilent, Santa Clara, CA) with the xCELLigence RTCA instrument (Roche). The plates were assembled according to the manufacturer´s instructions with complete medium (10% FBS) added to the bottom chambers. Cells were serum-starved for 4 h, detached using EDTA, washed in 1x PBS, counted and seeded in serum-free medium into the upper chambers at a density 7.5 × 10^4^ cells per well. Impedance was recorded every 15 min over a 12-hour period.

### Metastatic assay in vivo

The immunodeficient mice NOD.Cg-Prkdcscid Il2rgtm1Wjl/SzJ (NSG) were purchased from Charles River (Sulzfeld, Germany) and housed in compliance with ARRIVE guidelines. A total of 1 × 10^6^ SAOS-LM5 control and *CKB* KO cells (pool of both clones, 11B and 6C, mixed 1:1) suspended in 100 µl of 1x PBS were injected intravenously into the tail vein of 6–8 weeks old NSG mice. After 10 weeks, surface lung metastases were evaluated. All animals were sacrificed, the lungs were excised and fixed in Bouin’s solution (Sigma-Aldrich). Visible surface metastatic nodules were counted independently by two persons. Subsequently, mouse lungs tissue samples were manually sectioned on microtome at 4 μm, mounted on positively charged slides and then processed using an automated hematoxylin and eosin stainer. All procedure involving animals were approved by the Expert Committee for Ensuring the Welfare of Experimental Animals of Masaryk University and Ministry of Education, Youth and Sports of the Czech Republic (MSMT-6494/2023-3) and conducted by certified persons (JV, LK).

### Proteomic analysis

1 × 10^6^ SAOS-LM5 scrambled and *CKB* KO cells were cultured in three biological replicates. The cells were incubated for 48 h, washed three times with ice-cold 1x PBS and then lysed in 200 µl of the lysis buffer (8 M urea in 0.5 M triethylammonium bicarbonate (TEAB), pH 8.5, (both Sigma Aldrich), 1% phosphatase inhibitor cocktail (Thermo Fisher Scientific)). Sample preparation for proteomic profiling with the Filter Aided Sample Preparation (FASP) digestion on Microcon filter device (30 kDa cut-off; Millipore, Billerica, MA) and peptide desalting on BioPureSPN Midi C18 columns (Nest Group, Ipswich, MA) was performed as described previously [17] with the following modifications: (i) a total of 200 µg of protein material per sample was added to the Microcon filters, (ii) the protein samples were reduced with 20 µl of 100 mM tris-(2-carboxyethyl)phosphine in 100 µl of 8 M urea in 0.5 M TEAB, pH 8.5, (iii) the samples were alkylated with 20 µl of 300 mM iodoacetamide in 100 µl of 8 M urea in 0.5 M TEAB, pH 8.5, (iv) 6.67 µL of the trypsin solution (1 µg/µl) (Promega) was added at a 1:30 trypsin-to-protein ratio to the filters with 100 µl of 0.5 M TEAB, pH 8.5.

### LC-MS/MS measurement

The peptide extraction into LC-MS vials and peptide concentration measurement were performed as described in [18] with the following modification: the polyethylene glycol was replaced by n-Dodecyl β-D-maltoside (DDM, final concentration 0.1%).

LC-MS/MS analyses were performed using the UltiMate 3000 RSLCnano system connected to the timsTOF Pro 2 mass spectrometer (Bruker). The tryptic digests were concentrated and desalted using a trapping column (Acclaim PepMap Neo C18, dimensions 300 μm ID, 5 mm long, 5 μm particles, Thermo Fisher Scientific) prior to LC separation. The trapping column was washed with 0.1% TFA, and the peptides were eluted into an analytical column (Aurora C18, 75 μm ID, 250 mm long, 1.7 μm particles, Ion Opticks, P/N AUR3-25075C18-CSI) using a linear gradient program (3–42% of mobile phase B; mobile phase A: 0.1% formic acid (FA) in water; mobile phase B: 0.1% FA in 80% acetonitrile (ACN)) using a separation gradient of 120 min with 200 nl/min flow rate. Both the trapping and analytical columns were equilibrated prior to the sample injection. The analytical column was placed inside the Column Toaster (Bruker), with the emitter end installed in the CaptiveSpray ion source (Bruker) according to the manufacturer’s instructions with the column temperature set to 50 °C.

The data-independent acquisition (DIA) mode was used to acquire the MSn data in the m/z range of 100–1700 and in the 1/k0 range of 0.6–1.4 V x s x cm^− 2^. The data dependent acquisition (DDA) test runs were performed to optimize the DIA window scheme that was calculated to cover 75% of the identified peptides in the DDA test runs in the m/z range, ignoring 12.5% from the bottom and top m/z range covered for the DIA method. Two steps for each parallel accumulation-serial fragmentation (PASEF) scan and cycle time of 100 ms locked to 100% duty cycle was used. The ion mobility window was optimized in a similar way to the m/z range, based on the number of peptides identified in DDA test runs. The split into two ion mobility steps was positioned at the midpoint of the overlapping ion mobility region between the two m/z ranges covered within a single PASEF scan. A description of the DIA window scheme can be found in Supplementary material 4.

### LC-DIA-MS/MS data processing

For the LC-DIA-MS/MS measurements, protein identification and quantification were performed using Spectronaut 19.0 software (Biognosys, Schlieren, Switzerland) with the directDIA approach. The analysis was performed using the human UniProt/SwissProt database (downloaded on 2024-01-24, 20,597 sequences). The precursor and experiment protein Qvalue cutoff was set to 0.01. For total proteome, peptides were included if identified with a Qvalue < 0.01 in at least 3 of 9 analyses (Qvalue percentile 0.33 setting). Global imputation strategy was used. Carbamidomethyl (C) was set as a fixed modification, and Acetyl (Protein N-terminus) and Oxidation (M) were set as variable modifications. Enzyme specificity was set to trypsin/P with a maximum of 2 missed cleavages and 5 variable modifications per peptide. The unpaired Student’s t-test in Spectronaut software was used for differential abundance testing, the criteria for differentially abundant proteins were set as follows: absolute log2 Fold Change (|log2FC|) > 0.58 and a q-value < 0.05.

### Proteomics data analysis

A Venn diagram illustrating significant dysregulated proteins (q-value < 0.05 and (|log2FC|) > 0.58) was generated using the InteractiVenn web-based visualization tool [19]. Gene sets encoding significantly downregulated proteins (q-value < 0.05 and log2FC < -0.58) in both *CKB* KO clones relative to scrambled controls were analyzed for functional enrichment using the g:Profiler tool [20]. The organism of interest was specified as *Homo sapiens* and the analysis included pathways from Gene Ontology Biological Process (GO: BP) database. The list of genes coding for all proteins identified in the whole proteome experiment was used as a background. The levels of significantly downregulated proteins (q-value < 0.05, log2FC < -0.58) in both *CKB* KO clones associated with cell migration (based on g:Profiler results) were normalized using the Z-score. The heatmap was constructed using the ClustVis online tool (no scaling was applied; hierarchical clustering of rows and columns was performed using Euclidean distance and average linkage) [21].

### Statistical analyses

Statistical evaluations were conducted using GraphPad Prism v6.07 (Graph-Pad Software, La Jolla, CA). Results are expressed as mean ± SD from at least three independent experiments. An unpaired t-test was used for comparisons, unless stated otherwise. In the case of qPCR data, statistical significance was assessed using ΔCt values.

## Results

### CKB is a downstream molecule of the transcription factor c-Myb

**Fig. 1:**
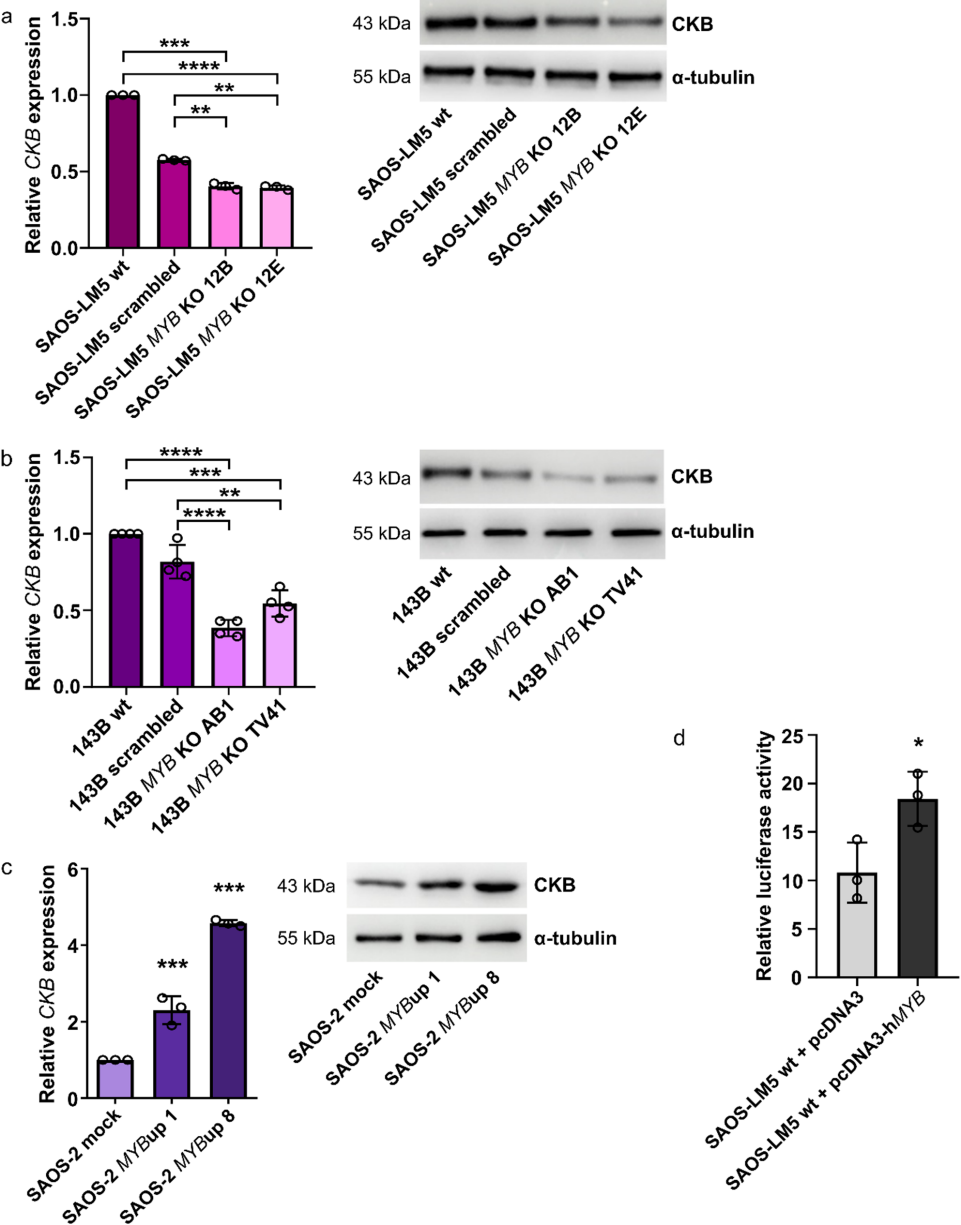
CKB is a downstream molecule of the transcription factor c-Myb. **a**,** b** Expression of *CKB*/CKB in control and SAOS-LM5 *MYB* KO (**a**) or 143B *MYB* KO (**b**) cells at mRNA and protein levels. **c** Expression of *CKB*/CKB in control and SAOS-2 *MYB*up cells at mRNA and protein levels. mRNA data were determined by real-time qPCR and represents mean ± SD of relative expression levels normalized to h*GAPDH*. Protein levels were determined by Western blot analysis. α-tubulin was used as a loading control. **d** Luciferase activity expressed in relative light units in SAOS-LM5 cells transiently co-transfected with pGL3-h*CKB*promoter reporter and pcDNA3 or pcDNA3-h*MYB* plasmids. Significant differences (*p < 0.05, **p < 0.01, ***p < 0.001, ****p < 0.0001) are indicated. Data represents mean ± SD from at least three independent experiments

We have recently identified *CKB* as one of the most downregulated genes in both 143B and SAOS-LM5 cells with knockout of the *MYB* gene in RNA-seq transcriptomic analysis [5]. To confirm a link between expression/activity of the c-Myb transcription factor and CKB, we first validated the decreased levels of both *CKB*/CKB mRNA and protein in these *MYB* KO cells (Fig. 1a, b). Next, we derived parental SAOS-2 cells with overexpression of *MYB*. Both SAOS-2 *MYB*up clones showed enhanced expression of *CKB*/CKB (Fig. 1c). Analysis of the *CKB* promoter sequence using the ConTra v3 tool [22] revealed the existence of a highly conserved MBS (c-Myb-binding site) at position −285 from TSS (transcription start site) (Supplementary Fig. 1). To confirm that c-Myb can transactivate *CKB*, we derived the reporter plasmid pGL3-h*CKB*promoter with the luciferase gene under the control of the 913 bp region of the *CKB* promoter. Transient co-transfection of SAOS-LM5 cells with pGL3-h*CKB*promoter reporter and pcDNA3-h*MYB* resulted in a 1.7-fold increase in luciferase activity compared to cells co-transfected with reporter and mock plasmids (Fig. 1d). These data indicate that CKB expression is regulated by the c-Myb in OSA cells. Subsequent experiments were performed to delineate the function of CKB in OSA.

### Selection of OSA cells with enhanced metastasis potential is associated with a cell line-dependent increase in CKB expression

**Fig. 2:**
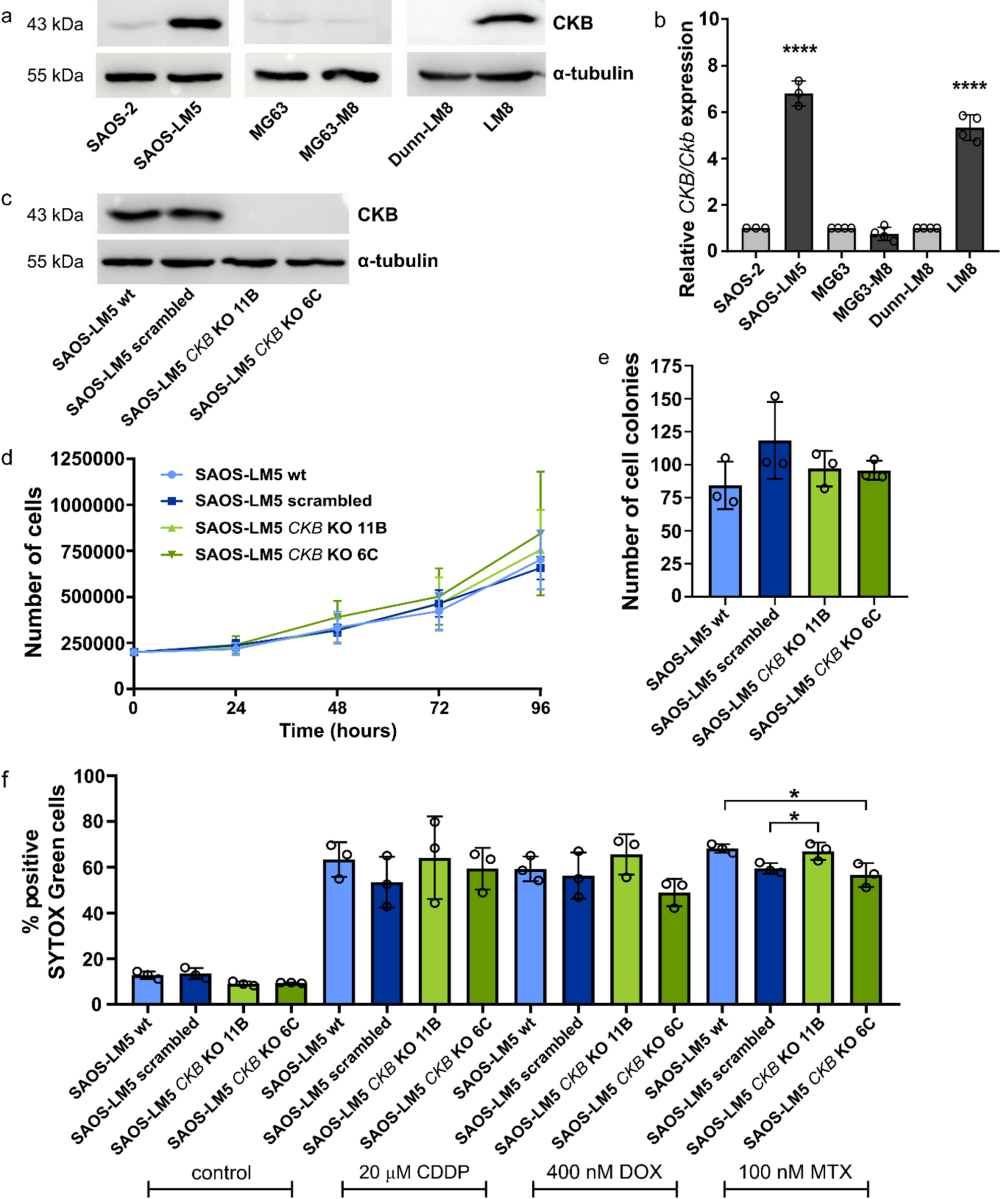
CKB expression is increased in metastatic counterparts of SAOS-2 and Dunn cell lines and its knockout does not alter proliferation and chemosensitivity of SAOS-LM5 cells. **a**,** b ***CKB* expression in pairs of parental (SAOS-2, MG63, Dunn) and highly metastatic (SAOS-LM5, MG63-M8, Dunn-LM8) OSA cell lines at protein (**a**) and mRNA (**b**) levels. mRNA data were determined by real-time qPCR and represents mean ± SD of relative expression levels normalized to h*GAPDH*/m*Gapdh*. Protein levels were determined by Western blot analysis. α-tubulin was used as a loading control. **c** Protein expression of CKB in SAOS-LM5 wt, scrambled and two independent SAOS-LM5 *CKB* KO clones (11B and 6C ). **d** Growth curve of SAOS-LM5 wt, scrambled and SAOS-LM5 *CKB* KO cells. **e** Number of cell colonies of SAOS-LM5 wt, scrambled and SAOS-LM5 *CKB* KO cells after 14 days of cultivation. **f** Sensitivity of SAOS-LM5 wt, scrambled and SAOS-LM5 *CKB* KO cells to LD_50_ of chemotherapeutics (CDDP, DOX and MTX) analyzed after SYTOX™ Green staining by flow cytometry. Significant differences (*p < 0.05, ***p < 0.0001) are indicated. Data represents mean ± SD from at least three independent experiments

c-Myb has previously been associated with metastasis in OSA [5]. To clarify the role of CKB in OSA metastasis, we first analyzed the expression of CKB in the parental OSA cell lines SAOS-2, MG63 and Dunn as well as in their metastatic derivatives SAOS-LM5, MG63-M8 and Dunn-LM8. Increased expression of *CKB* was observed in the metastatic SAOS-LM5 and Dunn-LM8 cells at both mRNA and protein levels (Fig. 2a, b), whereas no difference was found between MG63 and MG63-M8 cells, suggesting that the increase in *CKB* expression during the process of in vivo selection of metastatic cells is cell line-dependent.

### *CKB* knockout does not alter proliferation or chemosensitivity of OSA cells in vitro

We derived SAOS-LM5 *CKB* KO cells using the CRISPR/Cas9 approach. Two independent *CKB* KO single cell clones were generated, and the absence of CKB protein was confirmed by Western blot analysis (Fig. 2c). In addition, the presence of a short insertion/deletion within the *CKB* gene was validated by DNA sequencing.

We subsequently determined the proliferation and clonogenic capacity of *CKB* KO and control cells. *CKB* KO did not alter the growth rate and the colony-forming capacity of SAOS-LM5 cells (Fig. 2d, e; Supplementary Fig. 2). The sensitivity of SAOS-LM5 *CKB* KO cells to cisplatin (CDDP), doxorubicin (DOX) and methotrexate (MTX), drugs commonly used in OSA therapy [23], was also not altered (Fig. 2f).

### *CKB* knockout/inhibition reduces migration of OSA cells in vitro

**Fig. 3:**
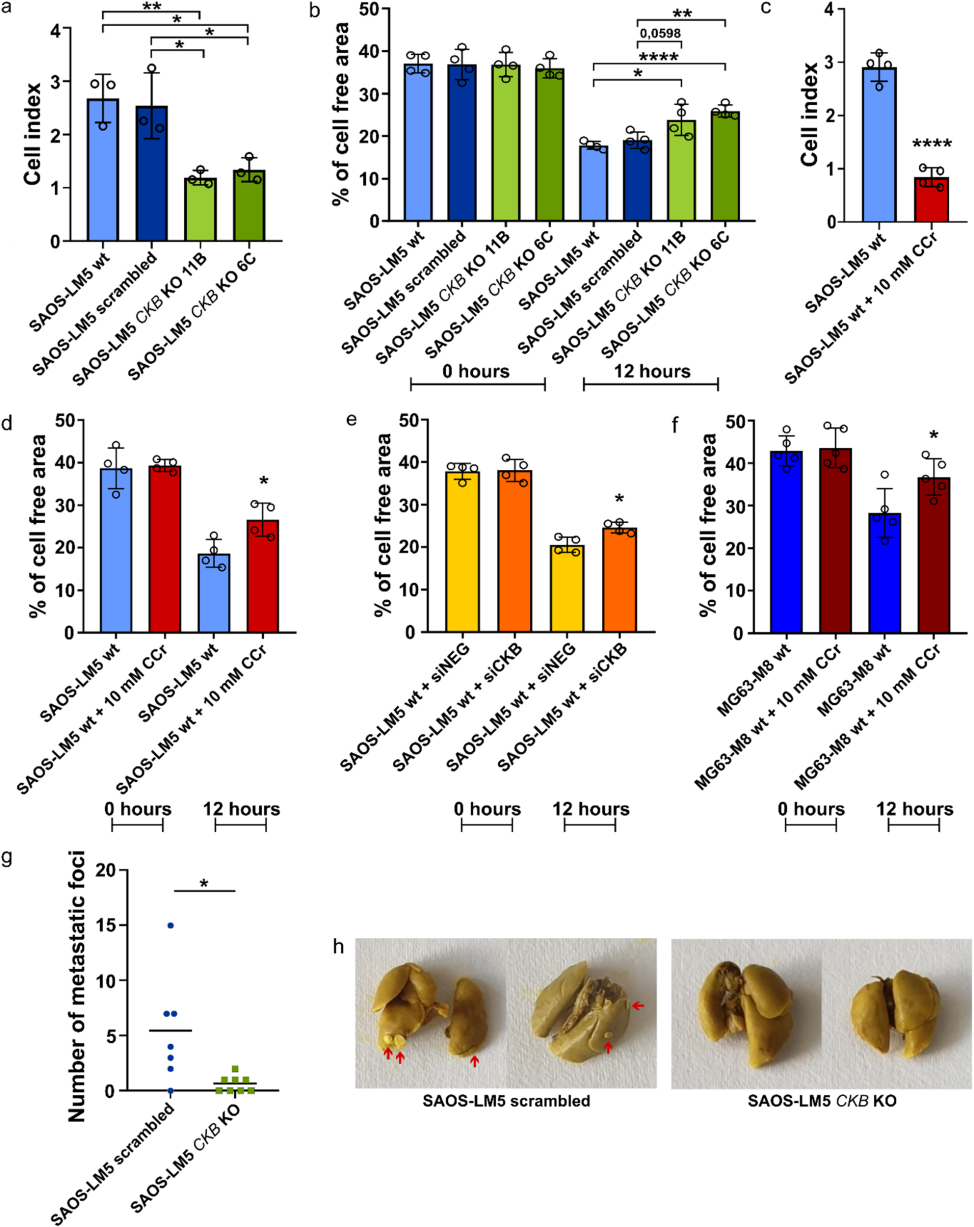
CKB inhibition suppresses migration and metastasis of OSA cells. **a**,** b** Migration of SAOS-LM5 wt, scrambled and SAOS-LM5 *CKB* KO cells evaluated using xCELLigence RTCA instrument (Roche) 6 h after cell seeding (**a**) or analyzed by scratch assay after 12 h (**b**). **c**,** d** Migration of SAOS-LM5 wt and SAOS-LM5 wt cells treated with 10 mM CCr analyzed using xCELLigence RTCA instrument (Roche) 6 h after cell seeding (**c**) or by scratch assay after 12 h (**d**). **e** Migration of SAOS-LM5 wt cells after siRNA transfection analyzed by scratch assay after 12 h. **f** Migration of control MG63-M8 wt and cells treated with 10 mM CCr analyzed by scratch assay after 12 h. Significant differences (*p < 0.05, **p < 0.01, ***p < 0.0001) are indicated. Data represents mean ± SD from at least three independent experiments. **g**,** h** SAOS-LM5 scrambled and SAOS-LM5 *CKB* KO cells were injected into the tail vein of NSG mice. Mice were sacrificed 10 weeks later, lungs were excised, fixed in Bouin’s solution and surface metastatic nodules were counted. Significant differences (^∗^*p* < 0.05) are indicated. Data represents a pool of two independent experiments

Next, we examined the effect of *CKB* KO on the motility of OSA cells. Analysis of cell migration revealed reduced migratory ability of *CKB* KO cells compared to controls determined by xCELLigence RTCA (Fig. 3a; Supplementary Fig. 3a) and scratch assay (Fig. 3b; Supplementary Fig. 4a). To confirm the involvement of CKB in the regulation of cell migration, we investigated the effect of CKB inhibition by CCr or siRNA. Both CCr treatment and siRNA transfection reduced cell migration of SAOS-LM5 (Fig. 3c, d,e; Supplementary Fig. 3b, 4b, c, 5) and MG63-M8 cells (Fig. 3f; Supplementary Fig. 4d, 5), confirming CKB as a positive regulator of OSA cell migration.

### *CKB* knockout reduces the metastatic capacity of OSA cells in vivo

To investigate the role of CKB in metastasis in vivo, scrambled and SAOS-LM5 *CKB* KO cells were injected into the tail vein of NSG mice, and the mice were sacrificed 10 weeks later. *CKB* KO cells exhibit a reduced capacity to form lung metastases compared to scrambled cells (Fig. 3g, h; Supplementary Fig. 6).

### Proteins involved in cell migration are altered in the proteomic screen of *CKB* KO cells

**Fig. 4:**
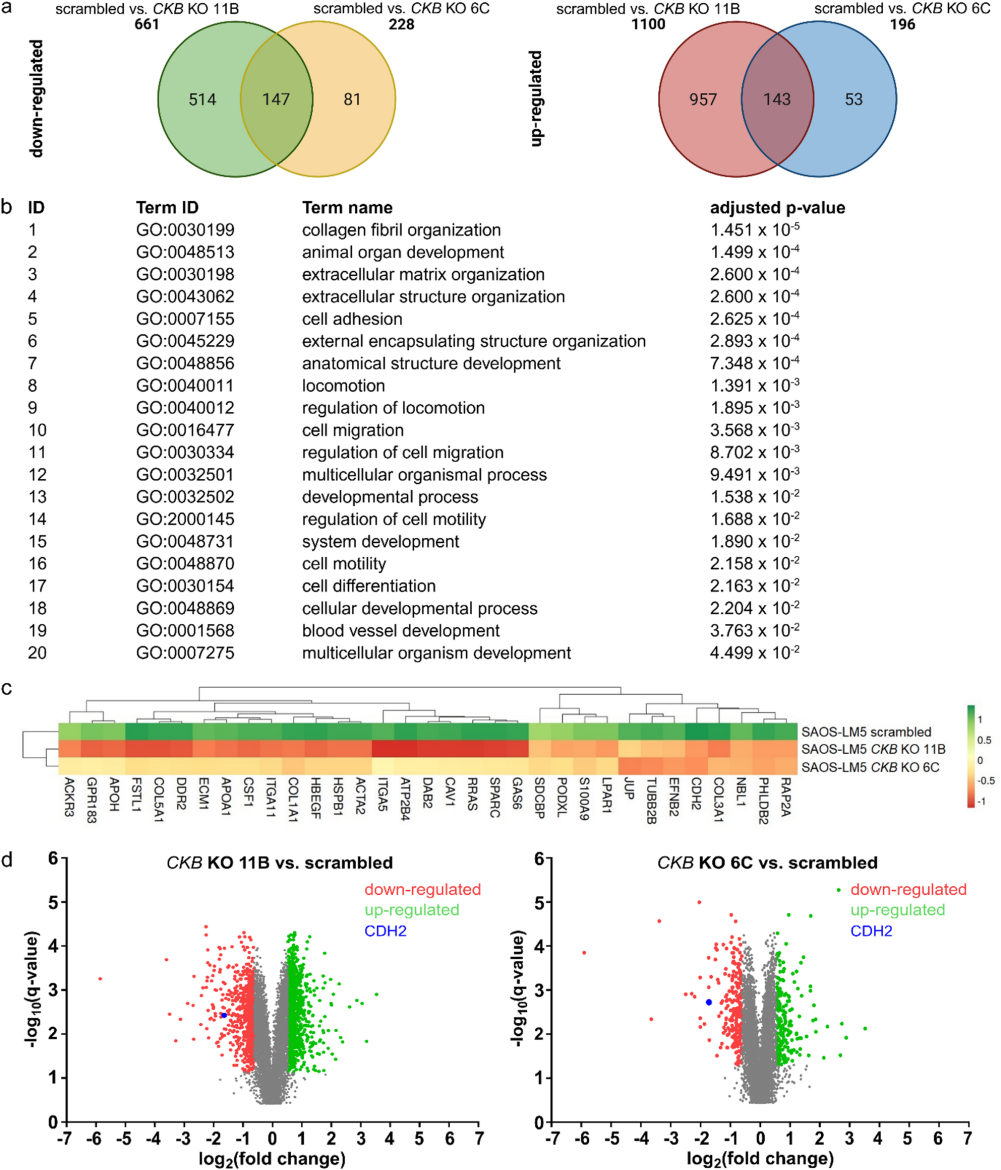
*CKB* depletion is associated with cell migration pathway in proteomic screen. **a** Overlap of down- and upregulated (q-value < 0.05 and (|log2FC|) > 0.58) proteins in SAOS-LM5 scrambled and *CKB* KO clones. **b** Statistically significant (adjusted p-value < 0.05) biological process pathways in g:Profiler GO analysis of 147 downregulated proteins in both *CKB* KO clones compared to scrambled control. **c** Heatmap of individual samples and protein groups clustering of SAOS-LM5 scrambled, and *CKB* KO clones according to the sample protein group quantity profile. **d** Volcano plots of differential protein abundance analysis between *CKB* KO clones and scrambled cells with N-cadherin (CDH2) highlighted

To identify proteins associated with *CKB* depletion, we performed proteomic analysis of SAOS-LM5 scrambled and SAOS-LM5 *CKB* KO clones. A total of 8474 protein groups (FDR = 0.01) were quantified using LC-MS/MS analysis in the diaPASEF mode (Supplementary material 5). This analysis showed 290 differentially abundant (q-value < 0.05, (|log2FC|) >0.58) proteins (147 downregulated and 143 upregulated) simultaneously in both SAOS-LM5 *CKB* KO cell lines compared to the SAOS-LM5 scrambled control cell line (Fig. 4a, Supplementary material 6). Gene Ontology (GO) pathway analysis using the g:Profiler tool [20] showed that the group of 147 proteins that were downregulated in both SAOS-LM5 *CKB* KO clones compared to the scrambled control cell line (Fig. 4a) were significantly (adjusted p-value < 0.05) associated with 20 GO biological processes (Fig. 4b; Supplementary material 7), including cell migration, cell motility and locomotion. The pathway associated with cell migration included 33 proteins that were downregulated in both SAOS-LM5 *CKB* KO clones compared to scrambled cells (Supplementary material 8). One of the most downregulated proteins in both clones, based on log2FC values, was N-cadherin (CDH2) (Fig. 4c; Supplementary material 6). GO pathway analysis performed using another tool, PANTHER [24], also identified cell migration among the enriched GO terms (Supplementary Fig. 7). Next, significantly altered proteins (q-value < 0.05) with more than 1.5-fold increase (log2FC > 0.58) or a more than 1.5-fold decrease (log2FC < -0.58) in abundance were visualized using volcano plots (Fig. 4d).

### *CKB* depletion reduces cell migration by downregulation of N-cadherin

**Fig. 5:**
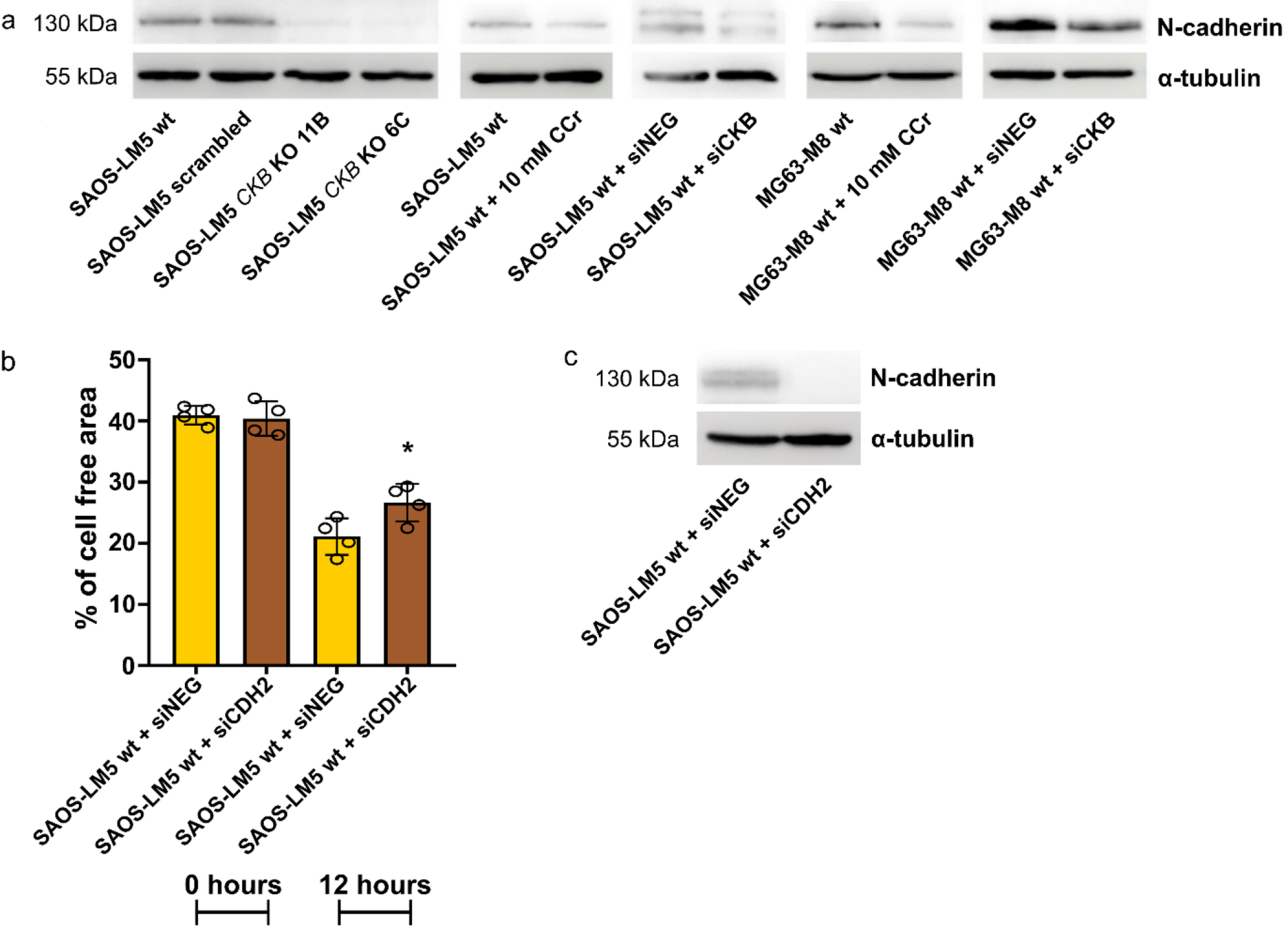
*CKB* depletion/inhibition reduces cell migration via N-cadherin. **a** N-cadherin protein expression in SAOS-LM5 wt, scrambled and SAOS-LM5 *CKB* KO clones, SAOS-LM5 wt and MG63-M8 wt cells treated with 10 mM CCr and after siRNA transfection. α-tubulin was used as a loading control. **b** Migration of SAOS-LM5 wt cells after siRNA transfection analyzed by scratch assay after 12 h. **c** Protein expression of CDH2 after siRNA transfection. α-tubulin was used as a loading control

Proteomic analysis revealed decreased protein levels of mesenchymal marker N-cadherin (CDH2) in both SAOS-LM5 *CKB* KO clones (Fig. 4c, d). The reduced expression of N-cadherin in SAOS-LM5 *CKB* KO compared to control cells was confirmed by Western blot analysis (Fig. 5a). Downregulation of N-cadherin was also observed in SAOS-LM5 wt and MG63-M8 wt cells after CCr treatment and siRNA transfection, respectively (Fig. 5a). To confirm the involvement of N-cadherin in the regulation of OSA cell migration, we transfected SAOS-LM5 wt cells with siRNA against *CDH2*. Subsequent scratch assay analysis revealed slower migration of *CDH2* knock-down cells compared to cells transfected with negative control siRNA (Fig. 5b, c; Supplementary Fig. 4e). These data strongly indicate that depletion/inhibition of CKB is associated with reduced levels of the mesenchymal marker N-cadherin and decreased cell migration.

## Discussion

We have recently shown that c-Myb acts as a metastasis promoter in OSA, and *CKB* was identified by transcriptomic analyses as one of the most downregulated genes in 143B and SAOS-LM5 *MYB* knockout OSA cells, which are characterized by a reduced metastatic capacity [5]. To date, only limited data are available on the functional role of CKB in OSA. Zhou et al. reported that CKB modulates proliferation, migration, invasion, and survival of OSA cell lines via the p53–p21 signaling pathway [10]. The authors analyzed cell phenotypes after transient transfection with a CKB-expressing plasmid or siRNA. However, in our hands, depletion of *CKB* by CRISPR/Cas9 had no effect on the proliferation of OSA cells. Moreover, p21 level was decreased in *CKB*-depleted cells (Supplementary Fig. 8a) while Zhou et al. reported an increase of p21 in siRNA-treated cells. Interestingly, transient downregulation of *CKB* with siRNA also leads to upregulation of p21 and reduced proliferation in our hands (Supplementary Fig. 8a, 9). An increase in p21 and a reduced proliferation rate have also been previously reported after *CKB* siRNA transfection in ovarian cancer cells [25]. This suggests that cells with depleted/inhibited *CKB*/CKB may initially respond with p21 upregulation and reduced proliferation but later adapt to these conditions by lowering p21 levels and restoring high proliferative capacity. Several mechanisms are possible for this adaptation. Other isoforms of CK have been described in human cells and their overexpression could compensate for the lack of CKB [7]. Similarly, other energy buffering and transfer systems, such as the adenylate kinase-mediated phosphotransfer pathway, may also step in to compensate for *CKB* depletion [26, 27].

SAOS-LM5 cells are considered p53-null, which we were able to confirm by Western blot analysis (Supplementary Fig. 8b) [28]. We thus hypothesize that a different pathway may link CKB expression/activity to p21. Previously, deregulation of p21 has been observed in cell lines with deleted p53, thus confirming the existence of other mechanisms of p21 regulation [29]. Since we observed reduced cell migration in cells with both long-term and transient CKB depletion, it appears that signaling pathways beyond p21 are involved.

Our proteomic analysis revealed that proteins involved in cell adhesion and migration are deregulated upon *CKB* depletion, including N-cadherin. N-cadherin was downregulated in *CKB* KO cells, as well as after transient transfection of *CKB* siRNA and CCr treatment. The downregulation of N-cadherin may thus explain the reduced cell migration observed under all three conditions. N-cadherin has already been identified as a target of numerous signalling pathways that modulate migration/invasion of OSA cell lines [30–32], and its upregulation has been associated with an EMT-like phenotype and enhanced migration in OSA [33]. At the same time, metastases occurred earlier in patients with higher expression of *CDH2* as revealed by an analysis of the available OSA transcriptomic dataset GSE21257 (Supplementary Fig. 10) [34]. The functional role of N-cadherin in the regulation of OSA cell migration was also confirmed by us in the knockdown experiment. While our in vitro and in vivo data support a functional link between CKB and N-cadherin in the regulation of OSA metastasis, we acknowledge that in vivo rescue experiments, such as re-expression of CKB/N-cadherin in CKB-deficient cells followed by metastasis assessment, were not performed. This represents a limitation of the study, as such experiments would provide more definitive evidence for the causal role of CKB in promoting metastasis via N-cadherin.

Interestingly, several other membrane proteins have been identified previously as interaction partners of CKB, including PCDHB10, a member of the cadherin superfamily [35]. CKB also colocalizes with E-cadherin in colon cancer cells and is important for the assembly of the adherens junction and the maintenance of epithelial integrity [36]. Previous studies have also linked CKB with cell migration by regulating the dynamics of the actin cytoskeleton. CKB co-localizes with F-actin in peripheral cellular structures, supports the formation of actin-based protrusions and regulates actin remodelling, cell spreading and migration [35, 37–39]. Thus, CKB can modulate cell migration via different mechanisms that, in contrast to the regulation of cell proliferation in OSA cell lines, are not compensated by alternative pathways.

A study by Zhou et al. reported that CKB is highly expressed in OSA tumors compared to adjacent normal tissue and that its expression is higher in established OSA cell lines compared to immortalized human osteoblasts [10]. These data support the tumor-promoting function of CKB in OSA. Besides tumor-promotion, our results also indicate a possible role of CKB in regulating migration and metastasis of OSA cells. Immunohistochemical analysis of CKB expression in a small set of OSA tumors revealed large heterogeneity among patients (Supplementary Fig. 11a, Supplementary methods). Moreover, analysis of the available OSA transcriptomic dataset GSE21257 [34] showed a trend towards earlier occurrence of metastases in patients with high *CKB* expression (Supplementary Fig. 11b, Supplementary methods).

The tumor-promoting role of creatine kinases in various cancers was recognized a long time ago [7–11]. However, their use as therapeutic targets was limited due to the lack of selective inhibitors. Previous studies have frequently used creatine analogues, such as CCr, to inhibit creatine phosphotransfer [40–43], but their non-specific nature and low potency limit their therapeutic efficacy. Novel selective compounds are currently being developed and tested in preclinical studies [44, 45].

To our knowledge, this is the first study showing a functional link between the *MYB* oncogene, *CKB* and cell migration/metastasis. Interestingly, *CKB* was identified as one of the 50 most downregulated genes in K562 erythroleukemia cells after *MYB* knockdown with two different siRNAs [46]. This is consistent with our findings in OSA, as *CKB* was among the top hits in both 143B and SAOS-LM5 *MYB* KO cells [5]. The follow-up study by Lemma et al. performed ChIP-seq analysis to identify c-Myb–binding sites in K562 chromatin [47]. We re-analyzed their data with a focus on *CKB* and identified multiple c-Myb ChIP-seq peaks at *CKB* loci (see Supplementary Fig. 12). These data suggest that *CKB* is likely a direct target of the c-Myb protein, although further validation in OSA cells and investigation of the precise regulatory mechanism are warranted.

In conclusion, our study reveals a novel MYB - CKB - CDH2 pathway involved in the regulation of OSA cell migration and metastasis. Further studies with recently identified small molecule CKB inhibitors are warranted to confirm its suitability as a molecular target for OSA therapy.

## Supplementary Information

Below is the link to the electronic supplementary material.

Supplementary Figures

Supplementary Material 1 - 3

Supplementary Material 4

Supplementary Material 5

Supplementary Material 6

Supplementary Material 7

Supplementary Material 8

Supplementary Methods

Supplementary Material 9 - uncropped WB

## Data Availability

The raw diaPASEF mass spectrometry proteomics data as well as Spectronaut data analysis output have been deposited in the ProteomeXchange Consortium via the Proteomics Identifications (PRIDE) partner repository [48] (http://www.ebi.ac.uk/pride/archive/) with the dataset identifier PXD065263.
