# Supplementary material for: Creatine kinase B, a downstream effector of c-Myb, controls migration of osteosarcoma cells via regulation of N-cadherin
Source: Cancer Cell Int. 2025 Dec 5;26:5. doi: 10.1186/s12935-025-04087-0 (PMC12797693; doi:10.1186/s12935-025-04087-0)
Supplement: Supplementary file 1 — Supplementary Figures [file 12935_2025_4087_MOESM1_ESM.docx]

Supplementary Fig. 1
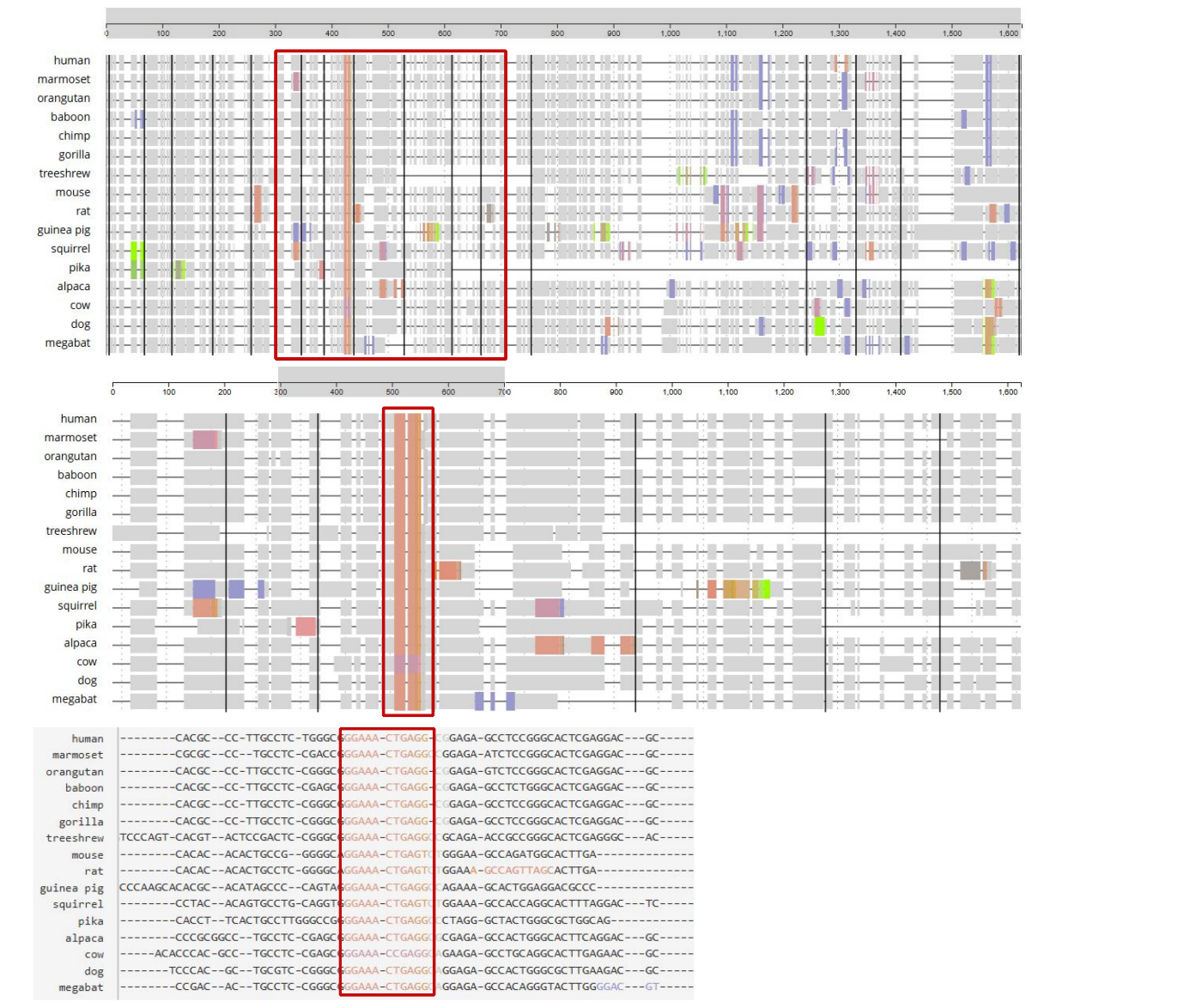


Supplementary Fig. 1: **Identification of a highly conserved c-Myb-binding site (MBS) at position -285 from TSS (transcription start site) using ConTra v3 tool** (Kreft *et al*. 2017).

Supplementary Fig. 2
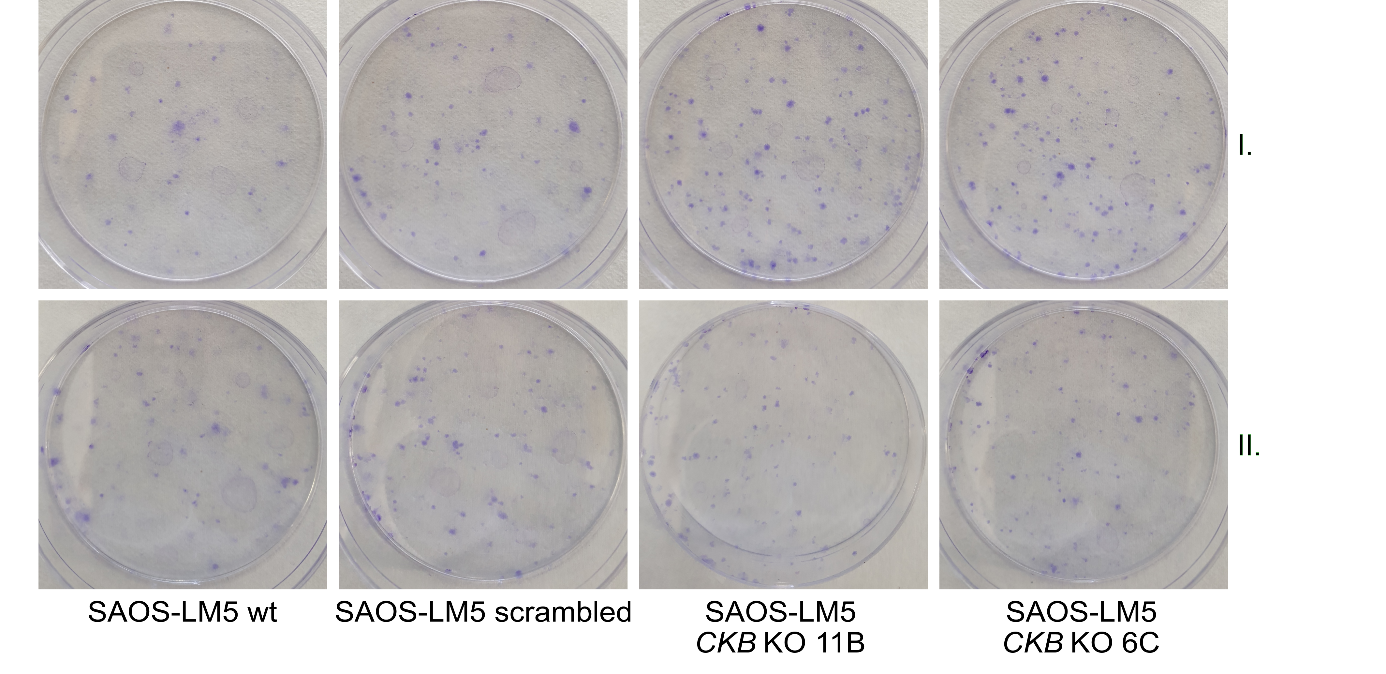


Supplementary Fig. 2: **Representative pictures of 2D cell colonies of SAOS-LM5 cells (wt, scrambled and *CKB* KO clones) after 14 days of cultivation**

Supplementary Fig. 3
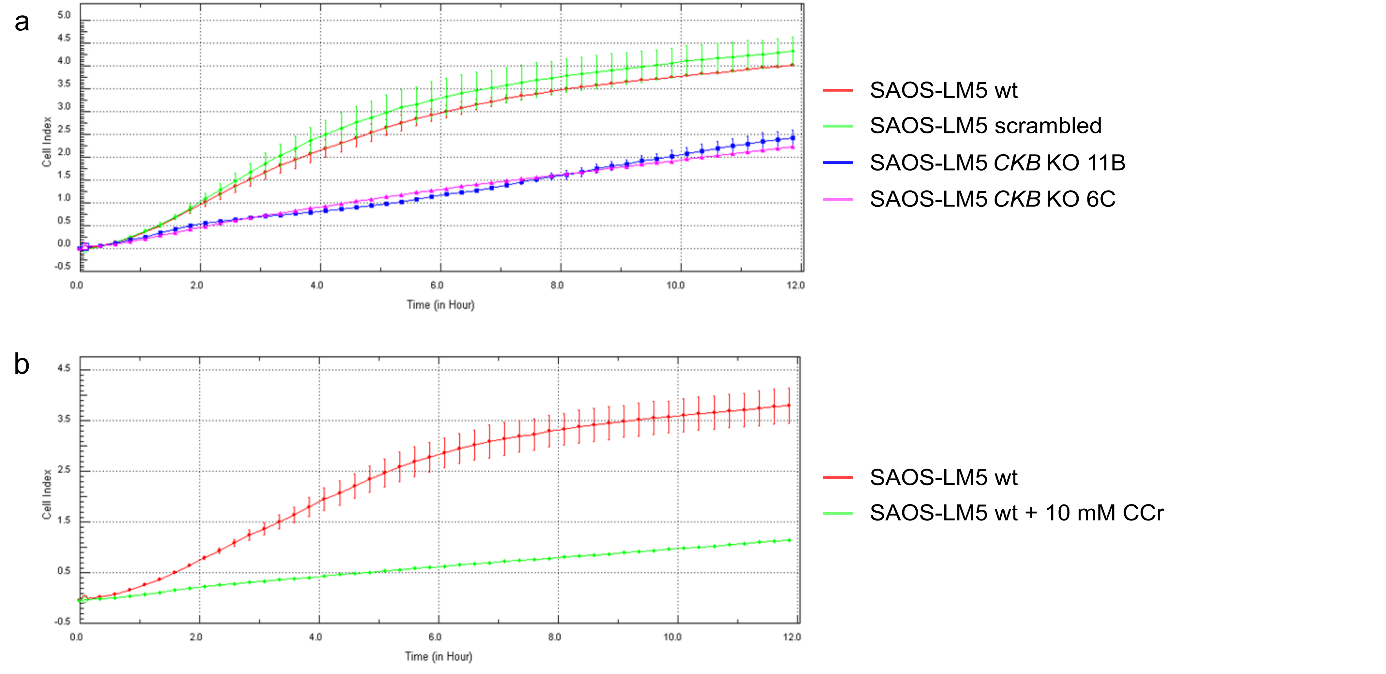


Supplementary Fig. 3: **Cell migration assessed by XCELLigence RTCA. a** Representative graph from XCELLigence RTCA showing real-time migration of SAOS-LM5 wt, scrambled and SAOS-LM5 *CKB* KO cells measured every 15 minutes for a period of 12 hours. **b** Representative graph from XCELLigence RTCA showing real-time migration of SAOS-LM5 wt and SAOS-LM5 treated with 10 mM CCr measured every 15 minutes for a period of 12 hours.

Supplementary Fig. 4
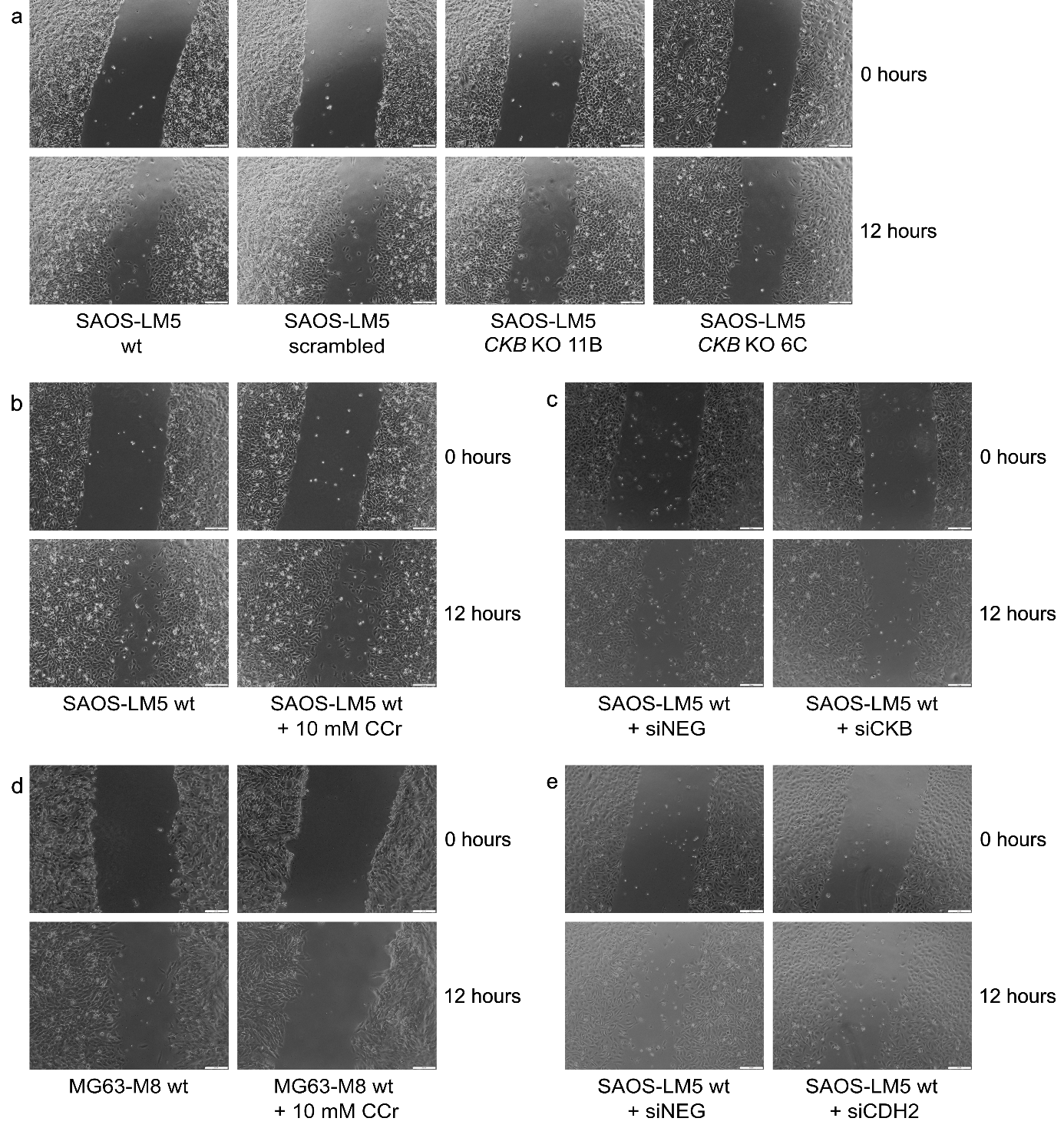


Supplementary Fig. 4: **Cell migration analyzed by scratch assay. a,b,c,d,e** Representative pictures of scratches at the beginning and after 12 hours obtained by light microscope showing migration of SAOS-LM5 wt, scrambled and SAOS-LM5 *CKB* KO cells (**a**), SAOS-LM5 wt and SAOS-LM5 wt cells treated with 10 mM CCr (**b**), SAOS-LM5 wt after siRNA transfection (**c,e**) and MG63-M8 wt and MG63-M8 wt cells treated with 10 mM CCr (**d**).

Supplementary Fig. 5
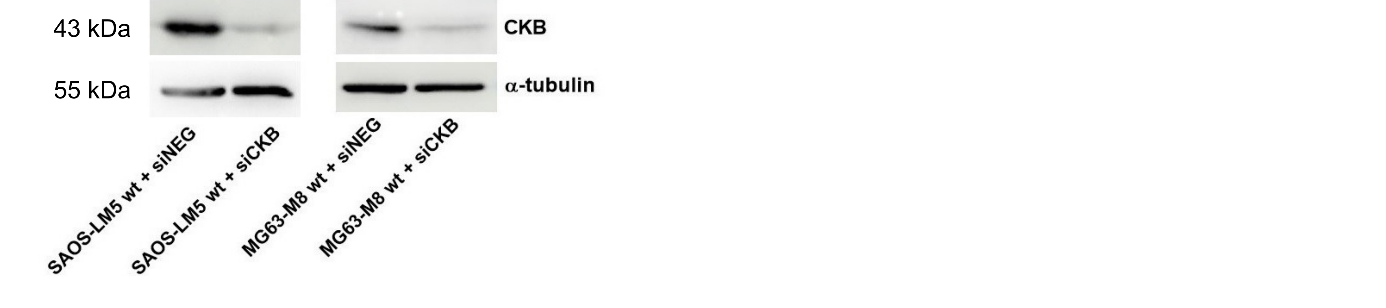


Supplementary Fig. 5: **Protein expression of CKB in OSA cell lines (SAOS-LM5 wt and MG63-M8 wt) after siRNA transfection. α‑tubulin was used as a loading control**

Supplementary Fig. 6


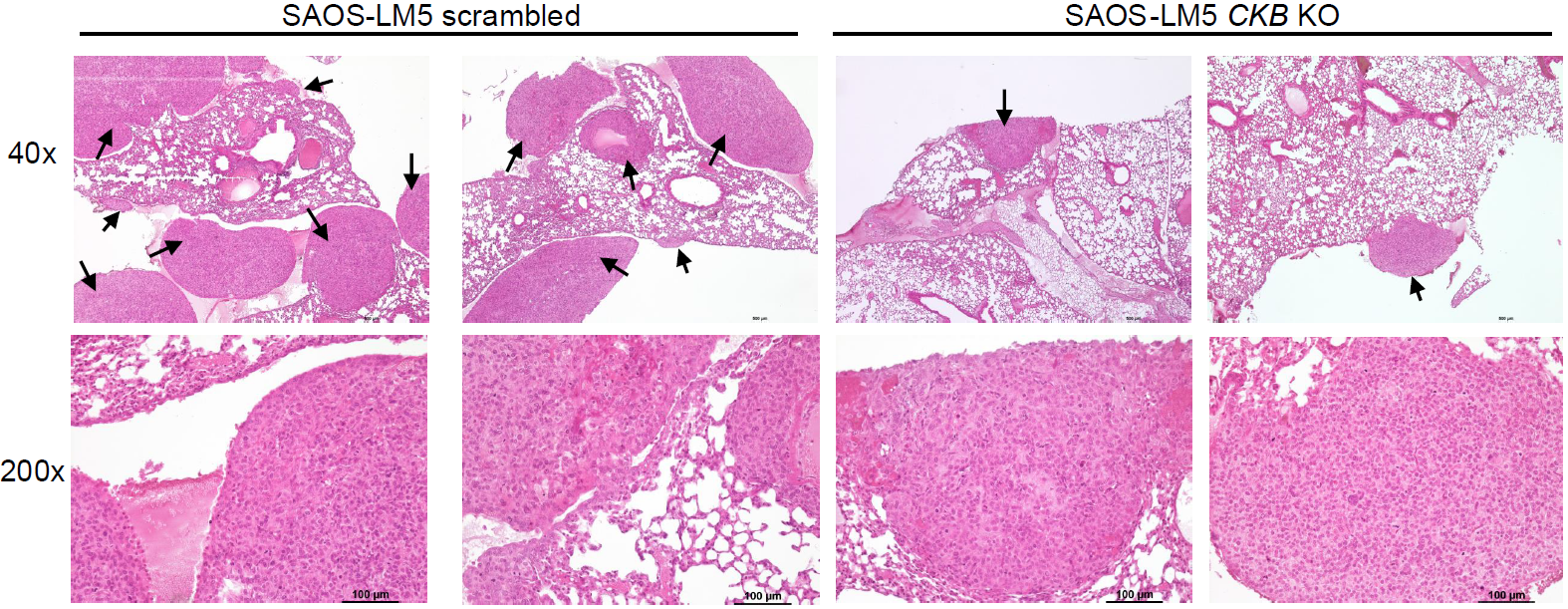


Supplementary Fig. 6: **Representative H&E stained sections of lungs from mouse injected with SAOS-LM5 scrambled and SAOS-LM5 *CKB* KO cells; scale bar = 500 and 100 μm.**

Supplementary Fig. 7
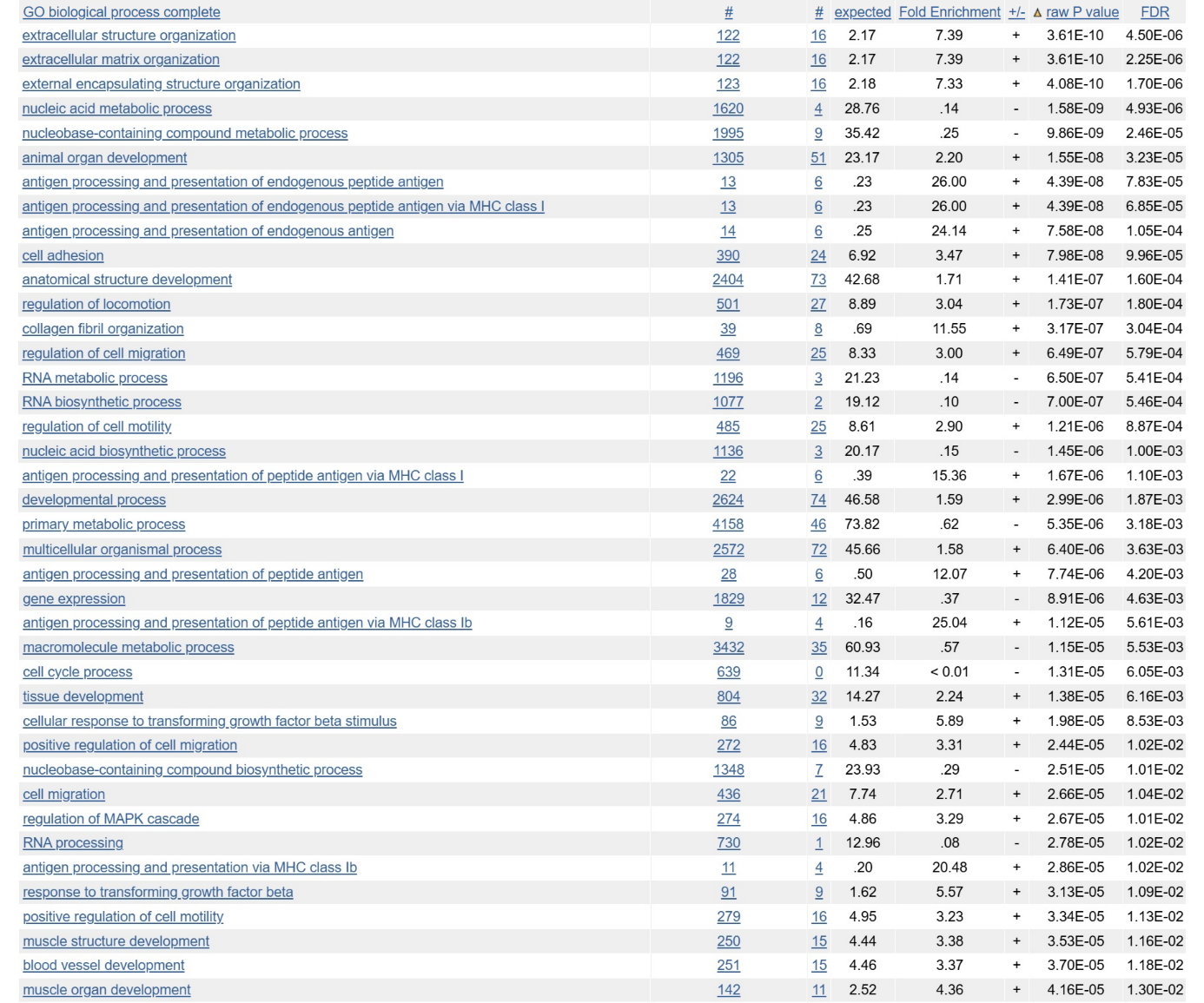


Supplementary Fig. 7: **GO analysis results from PANTHER.** TOP 40 statistically significant (FDR < 0.05) biological processes pathways in PANTHER GO analysis (Thomas *et al.* 2022) of 147 down-regulated proteins in both *CKB* KO clones compared to scrambled control.

Supplementary Fig. 8
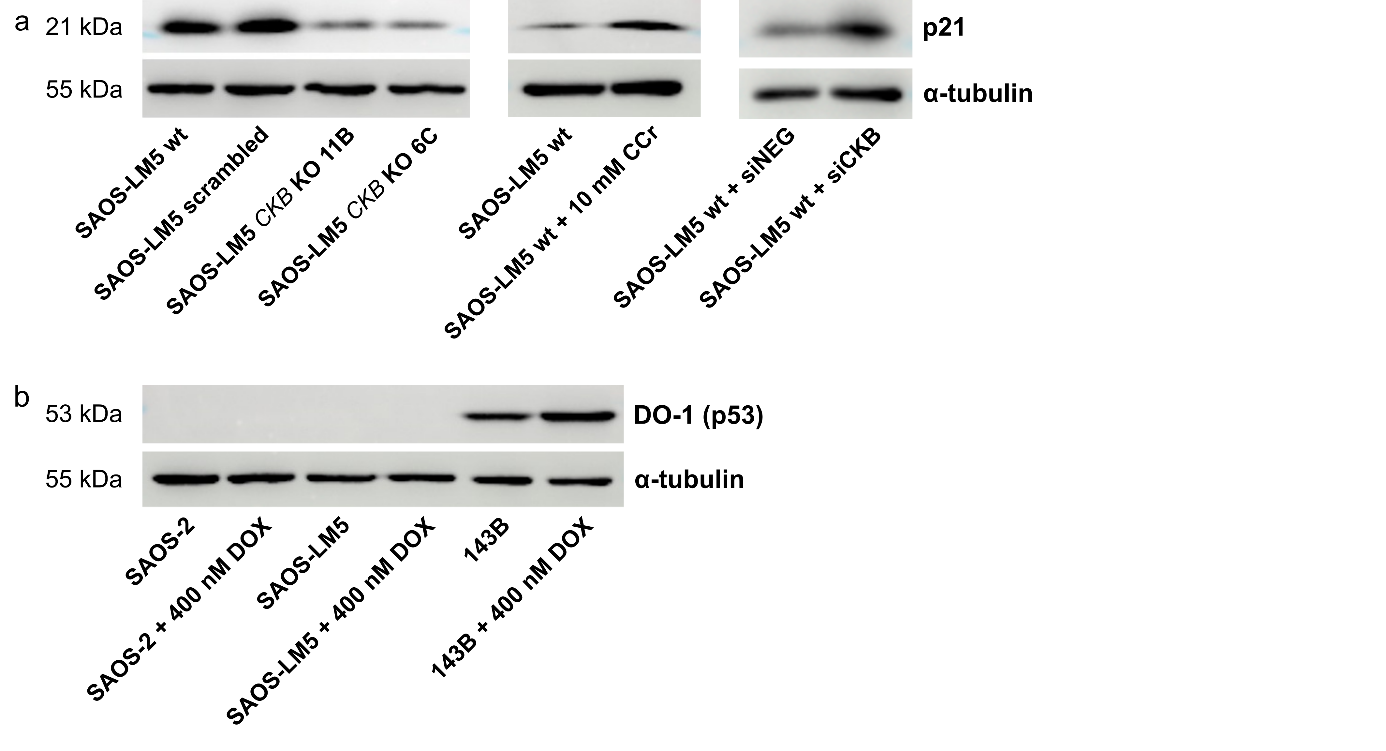


Supplementary Fig. 8: **Protein expression of p21 and p53 in OSA cell lines. a** Protein level of p21 in SAOS-LM5 wt, scrambled, *CKB* KO cells, and in SAOS-LM5 wt control cells and cells treated with 10 mM CCr or after siRNA transfection. **b** Protein level of p53 detected with DO-1 antibody in SAOS-2, SAOS-LM5 and 143B (p53-positive) cells and cells treated with doxorubicin (DOX) as inducer of DNA damage and p53 activation. α‑tubulin was used as a loading control.

Supplementary Fig. 9
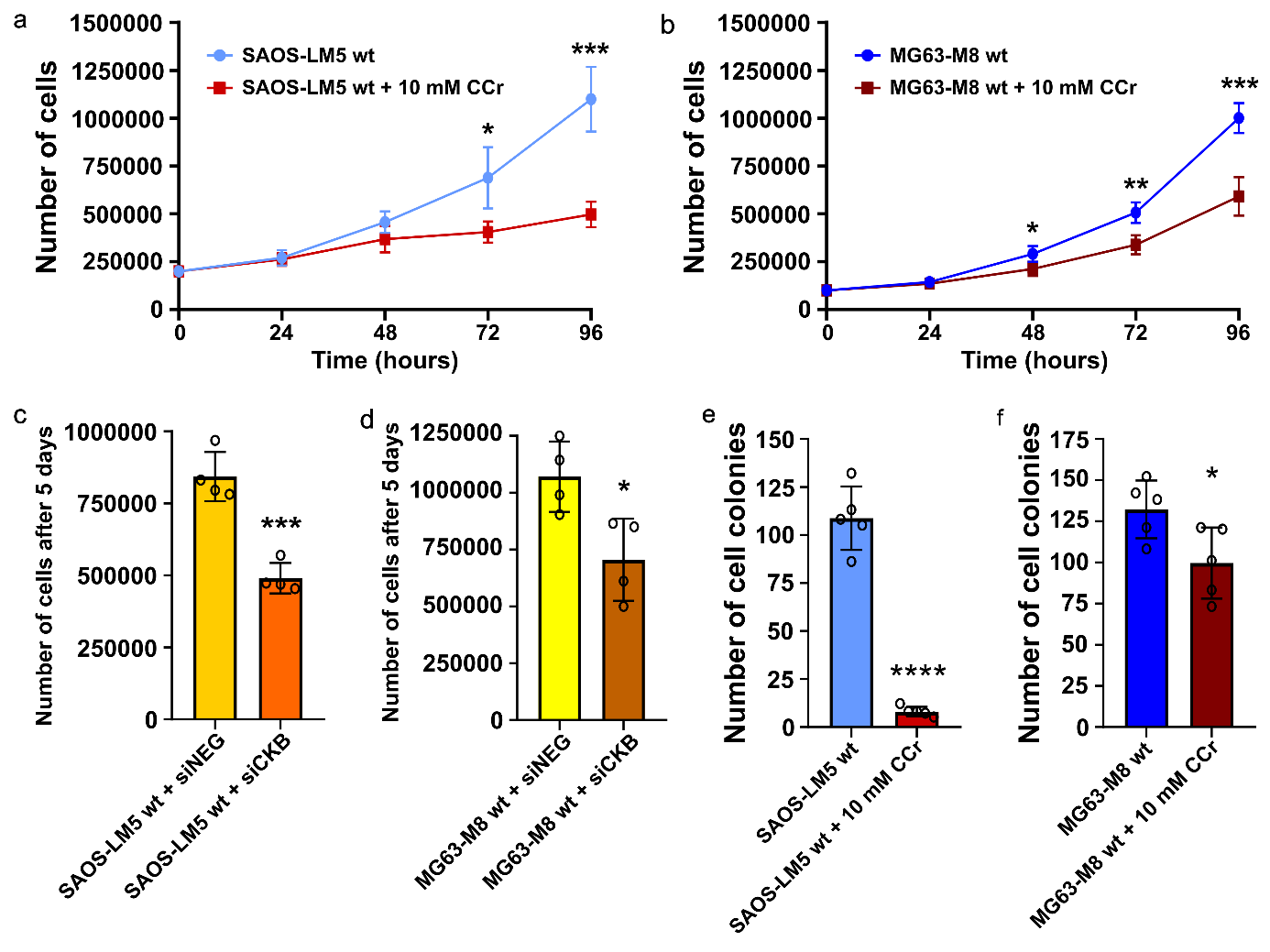


Supplementary Fig. 9: **CKB inhibition by CCr of siRNA decreased proliferation and colony forming ability of SAOS-LM5 and MG63-M8 cells. a,b** Growth curve of SAOS-LM5 wt (**a**) and MG63-M8 wt (**b**) cells treated with solvent or 10 mM CCr. **c,d** Number of siRNA-treated SAOS-LM5 wt (**c**) or MG63-M8 wt (**d**) cells after 5 days of cultivation. **e,f** Number of cell colonies of SAOS-LM5 wt (**e**) and MG63-M8 wt (**f**) cells treated with solvent or 10 mM CCr after 14 days of cultivation. Significant differences (∗ p<0.05, ∗∗ p<0.01, ∗∗∗ p<0.001, ∗∗∗∗ p<0.0001) are indicated. Data represents mean±SD from at least three independent experiments.

Supplementary Fig. 10
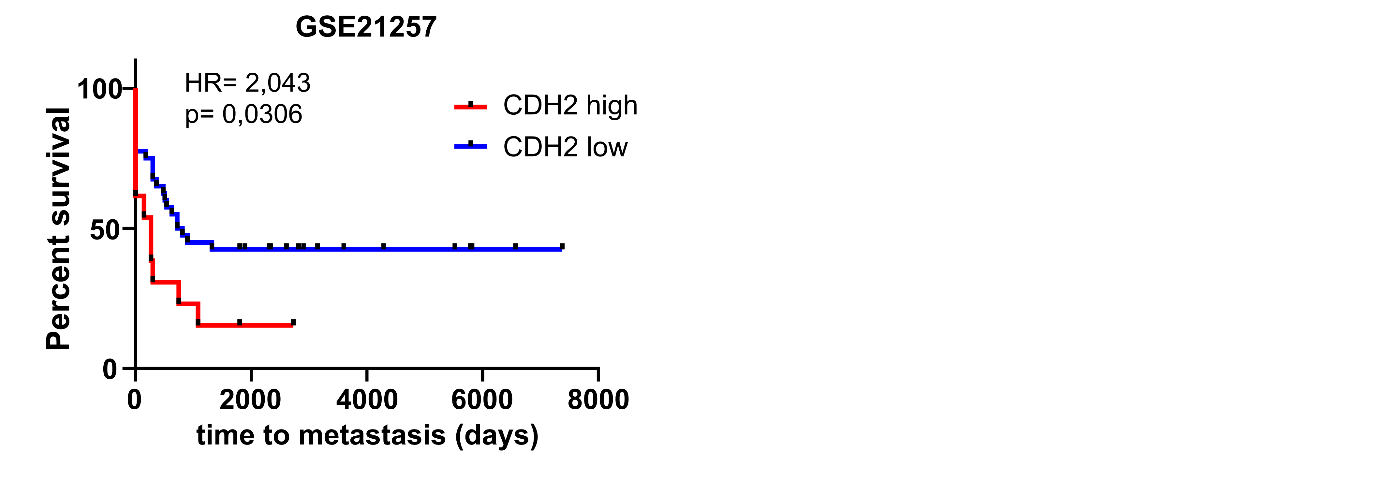


Supplementary Fig. 10: **Kaplan-Meier curve of time to metastasis development by CDH2 expression (data retrieved from GSE21257 dataset)** (Buddingh *et al*. 2011)**.**

Supplementary Fig. 11


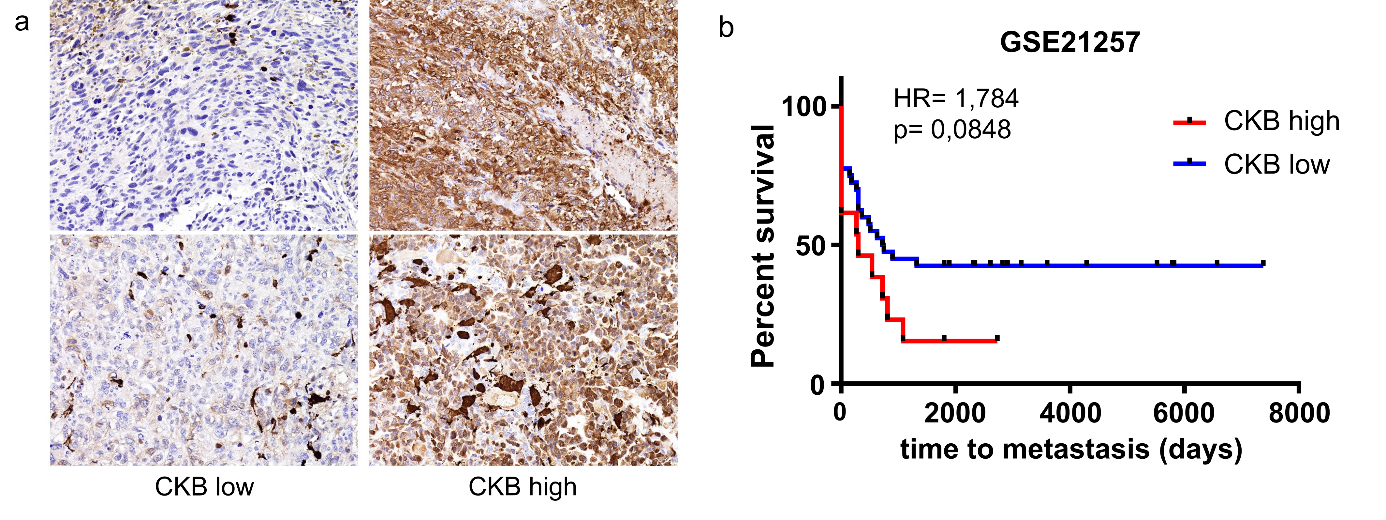


Supplementary Fig. 11: **CKB expression heterogeneity in OSA tumours tends to be associated with time from diagnosis to metastatic progression**. **a** Expression of CKB in human OSA tissues (IHC analysis of specimen with low (left) and high (right) level of CKB; original magnification ×200). **b** Kaplan-Meier curve of time to metastasis development by CKB expression (data retrieved from GSE21257 dataset) (Buddingh *et al*. 2011)**.**

Supplementary Fig. 12
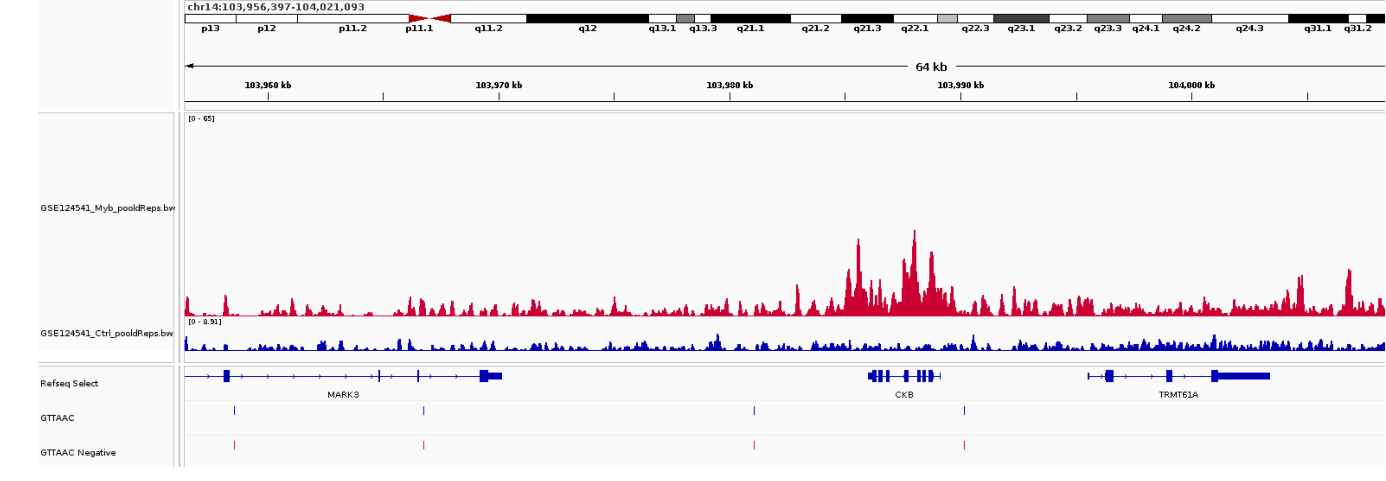


Supplementary Fig. 12: **ChIP-seq peaks in the vicinity of the *CKB* gene.** Figure shows ChIP-seq peaks from BigWig files provided by Lemma *et al*., (dataset GSE124541) that used c-Myb as bait in human K562 cells (Lemma *et al*. 2021). The two tracks viewed in the Integrated Genome Viewer (IGV) represent control (blue) and c-Myb input (red).

**References**

Buddingh EP, Kuijjer ML, Duim RA, Bürger H, Agelopoulos K, Myklebost O, Serra M, Mertens F, Hogendoorn PC, Lankester AC, Cleton-Jansen AM. Tumor-infiltrating macrophages are associated with metastasis suppression in high-grade osteosarcoma: a rationale for treatment with macrophage activating agents. Clin Cancer Res. 2011;17(8):2110-2119.

Kreft L, Soete A, Hulpiau P, Botzki A, Saeys Y, De Bleser P. ConTra v3: a tool to identify transcription factor binding sites across species, update 2017. Nucleic Acids Res. 2017;45:490-494.

Lemma RB, Ledsaak M, Fuglerud BM, Sandve GK, Eskeland R, Gabrielsen OS. Chromatin occupancy and target genes of the haematopoietic master transcription factor MYB. Sci Rep. 2021;11(1):9008.

Thomas PD, Ebert D, Muruganujan A, Mushayahama T, Albou LP, Mi H. PANTHER: Making genome-scale phylogenetics accessible to all. Protein Sci. 2022;31(1):8-22.
