# Supplementary material for: Creatine kinase B, a downstream effector of c-Myb, controls migration of osteosarcoma cells via regulation of N-cadherin
Source: Cancer Cell Int. 2025 Dec 5;26:5. doi: 10.1186/s12935-025-04087-0 (PMC12797693; doi:10.1186/s12935-025-04087-0)
Supplement: Supplementary file 2 — Supplementary Material 1 - 3 [file 12935_2025_4087_MOESM2_ESM.docx]

# **Supplementary material 1: Primers and oligonucleotides used for cloning**

CRISPR *CKB* 5ʹ - ACCGCGCGCTCAAGAGCATGACGG - 3ʹ

5ʹ - AAACCCGTCATGCTCTTGAGCGCGC - 3ʹ

CRISPR scrambled 5ʹ - CACCGAACAGTCGCGTTTGCGACTG - 3ʹ

5ʹ - AAACCAGTCGCAAACGCGACTGTTC - 3ʹ

*CKB* promoter 5ʹ - GAGCGGTACCCTTCCCGCATTCCAGCTCAA - 3ʹ

5ʹ - GAGCGAGCTCCGGCTCTTAAGGGGCACAA - 3ʹ

## **Supplementary material 2: Plasmids derived in this study**

pGL3‑h*CKB* promoter

vector backbone: pGL3 (Promega, E1751)

insert: human *CKB* promoter region was PCR amplified using primers “CKB promoter” (Supplementary material 1) from genomic DNA obtained from HUVEC cell line (amplicon 913 bp)

cloning: *Kpn*I + *Sac*I

pSpCas9(BB)‑2A‑GFP (PX458) scrambled GFP

vector backbone: pSpCas9(BB)‑2A‑GFP (PX458, Addgene)

insert: CRISPR scrambled sequence

cloning: *Bbs*I

pSpCas9(BB)‑2A‑GFP (PX458) CRISPR h*CKB*

vector backbone: pSpCas9(BB)‑2A‑GFP (PX458, Addgene)

insert: human *CKB* CRISPR sequence

cloning: *Bbs*I

pcDNA4/TO-h*MYB*

vector backbone: pcDNA4/TO (Invitrogen)

insert: *MYB* coding sequence from pcDNA3-h*MYB* (Dúcka *et al.* 2021)

cloning: *Kpn*I + *Xba*I

# **Supplementary material 3: Primers used for qPCR**

Human *CKB* 5ʹ - TCTGGCACAATGACAATAAGACC - 3ʹ

5ʹ - AAGAGAGTTTCAATCTGGGTGA - 3ʹ

Human *GAPDH* 5ʹ - TCGGAGTCAACGGATTTGGT - 3ʹ

5ʹ - TTCCCGTTCTCAGCCTTGAC - 3ʹ

Human *CDH2* 5ʹ - AGTCACCGTGGTCAAACCAA - 3ʹ

5ʹ - ACAGACACGGTTGCAGTTGA - 3ʹ

Mouse *Ckb* 5ʹ - TAGACAATCCGGGCCACC - 3ʹ

5ʹ - AGGTCGGTCTTGTGCTCATC - 3ʹ

Mouse *Gapdh* 5ʹ - ACTTGGCAGGTTTCTCCAGG - 3ʹ

5ʹ - TCATGACCACAGTCCATGCC - 3ʹ

**References**

Dúcka M, Kučeríková M, Trčka F, Červinka J, Biglieri E, Šmarda J, Borsig L, Beneš P, Knopfová L. c-Myb interferes with inflammatory IL1α-NF-κB pathway in breast cancer cells. Neoplasia. 2021;23(3):326-336.
