# Supplementary material for: Creatine kinase B, a downstream effector of c-Myb, controls migration of osteosarcoma cells via regulation of N-cadherin
Source: Cancer Cell Int. 2025 Dec 5;26:5. doi: 10.1186/s12935-025-04087-0 (PMC12797693; doi:10.1186/s12935-025-04087-0)
Supplement: Supplementary file 7 — Supplementary Material 8 [file 12935_2025_4087_MOESM7_ESM.docx]

|  |  |  | **scrambled vs.** *CKB* **KO 11B** | | **scrambled vs.** *CKB* **KO 6C** | |
| --- | --- | --- | --- | --- | --- | --- |
| **Gene name** | **UniProt ID** | **Protein description** | **log2 fold change** | **q-value** | **log2 fold change** | **q-value** |
| JUP | P14923 | Junction plakoglobin | 0.74 | 0.02331 | 1.01 | 0.01168 |
| SPARC | P09486 | SPARC | 1.19 | 0.00012 | 0.62 | 0.00088 |
| COL5A1 | P20908 | Collagen alpha-1(V) chain | 1.24 | 0.00044 | 0.72 | 0.00134 |
| ECM1 | Q16610 | Extracellular matrix protein 1 | 1.30 | 0.01084 | 0.84 | 0.01823 |
| RRAS | P10301 | Ras-related protein R-Ras | 1.24 | 0.00023 | 0.62 | 0.00082 |
| CSF1 | P09603 | Macrophage colony-stimulating factor 1 | 1.50 | 0.00160 | 0.89 | 0.00613 |
| DDR2 | Q16832 | Discoidin domain-containing receptor 2 | 1.20 | 0.00056 | 0.71 | 0.00110 |
| CDH2 | P19022 | Cadherin-2 | 1.61 | 0.00202 | 1.69 | 0.00266 |
| FSTL1 | Q12841 | Follistatin-related protein 1 | 1.92 | 0.00182 | 1.05 | 0.00314 |
| GPR183 | P32249 | G-protein coupled receptor 183 | 1.31 | 0.00517 | 0.63 | 0.00757 |
| DAB2 | P98082 | Disabled homolog 2 | 1.60 | 4.67x10^-5^ | 0.70 | 0.00128 |
| TUBB2B | Q9BVA1 | Tubulin beta-2B chain | 0.64 | 0.00206 | 0.77 | 0.00164 |
| PHLDB2 | Q86SQ0 | Pleckstrin homology-like domain family B member 2 | 1.14 | 0.00196 | 1.11 | 0.00352 |
| ATP2B4 | P23634 | Plasma membrane calcium-transporting ATPase 4 | 1.73 | 6.75x10^-5^ | 0.73 | 0.00023 |
| HBEGF | Q99075 | Proheparin-binding EGF-like growth factor | 1.96 | 0.00264 | 1.19 | 0.00863 |
| ITGA5 | P08648 | Integrin alpha-5 | 2.39 | 0.00067 | 0.75 | 0.00542 |
| HSPB1 | P04792 | Heat shock protein beta-1 | 1.47 | 0.00164 | 0.98 | 0.00236 |
| PODXL | O00592 | Podocalyxin | 1.87 | 0.00963 | 1.25 | 0.01845 |
| S100A9 | P06702 | Protein S100-A9 | 1.80 | 0.01168 | 1.42 | 0.03240 |
| COL1A1 | P02452 | Collagen alpha-1(I) chain | 3.11 | 0.00253 | 1.84 | 0.00734 |
| ACKR3 | P25106 | Atypical chemokine receptor 3 | 1.46 | 0.00191 | 0.73 | 0.02006 |
| APOA1 | P02647 | Apolipoprotein A-I | 1.05 | 0.00274 | 0.66 | 0.00781 |
| CAV1 | Q03135 | Caveolin-1 | 2.34 | 0.00043 | 0.92 | 0.00532 |
| COL3A1 | P02461 | Collagen alpha-1(III) chain | 0.96 | 0.00089 | 0.79 | 0.00163 |
| GAS6 | Q14393 | Growth arrest-specific protein 6 | 1.30 | 0.00014 | 0.68 | 0.00088 |
| LPAR1 | Q92633 | Lysophosphatidic acid receptor 1 | 1.04 | 0.00058 | 0.76 | 0.02750 |
| ITGA11 | Q9UKX5 | Integrin alpha-11 | 1.21 | 0.00050 | 0.79 | 0.00366 |
| NBL1 | P41271 | Neuroblastoma suppressor of tumorigenicity 1 | 0.99 | 0.00225 | 0.97 | 0.00972 |
| RAP2A | P10114 | Ras-related protein Rap-2a | 0.62 | 0.00381 | 0.59 | 0.00379 |
| EFNB2 | P52799 | Ephrin-B2 | 0.74 | 0.00287 | 0.86 | 0.00168 |
| SDCBP | O00560 | Syntenin-1 | 0.80 | 0.00954 | 0.69 | 0.01354 |
| ACTA2 | P62736 | Actin, aortic smooth muscle | 0.83 | 0.00046 | 0.58 | 0.00155 |
| APOH | P02749 | Beta-2-glycoprotein 1 | 1.70 | 0.00575 | 0.77 | 0.04689 |

**Supplementary file 8**. Proteins downregulated in SAOS-LM5 *CKB* KO 11B and 6C clones compared to control scrambled cells enriched in GO:BP cell migration pathway.
