# Supplementary material for: Creatine kinase B, a downstream effector of c-Myb, controls migration of osteosarcoma cells via regulation of N-cadherin
Source: Cancer Cell Int. 2025 Dec 5;26:5. doi: 10.1186/s12935-025-04087-0 (PMC12797693; doi:10.1186/s12935-025-04087-0)
Supplement: Supplementary file 8 — Supplementary Methods [file 12935_2025_4087_MOESM8_ESM.docx]

## **Supplementary methods**

## **Patients**

Four patients (two females aged 13 and 15 years and two males aged 15 and 16 years) diagnosed with high-grade OSA at the Department of Pediatric Oncology, University Hospital Brno participated in this study. All cases were diagnosed according to the current WHO Classification of Tumors of Soft Tissue and Bone (Anderson *et al*. 2021). Written informed consent was obtained from all patients, or their legal guardians and the study was approved by the Ethics Committee of the University Hospital Brno (21-080622/EK) and Masaryk University (EKV-2023-064). The study was conducted in accordance with the Declaration of Helsinki for research involving human participants. Only samples that did not require decalcification were selected as this procedure can interfere with protein detection and lead to false-negative results in immunohistochemical (IHC) detection (Miquelestorena-Standley *et al.* 2020). Tissue specimens were fixed in 10% neutral buffered formalin, embedded in paraffin and subsequently stained with hematoxylin-eosin and examined by pathologists with expertise in sarcomas. Representative tissue blocks were selected for IHC analysis. All tumor tissue samples were obtained prior to administration of chemotherapy.

## **Immunohistochemistry**

Formalin-fixed paraffin-embedded tissue blocks were sectioned at a thickness of 2 μm using a Leica SM 2010R microtome (Leica, Wetzlar, Germany) and mounted on positively charged TOMO Matsunami slides (Osaka, Japan). IHC was performed using an automated immunostainer (BenchMark ULTRA, Ventana Medical Systems, Tucson, AR). Antigen retrieval was performed with CC1 antigen retrieval solution pH 8.0, Ventana Medical Systems) for 20 minutes at 95 °C. Specimens were incubated with a primary antibody directed against CKB (rabbit monoclonal antibody, Clone ARC 1990, Invitrogen, Rockford, USA), at a concentration of 1:200 for 20 minutes at 36 °C, and then visualized using the Ultra View DAB IHC Detection Kit (Ventana Medical Systems). Subsequently, the specimens were counterstained with haematoxylin Gill (Merck, Darmstadt, Germany) for 1 minute, bluing in tap water, and coverslipped. Each IHC run contained a positive control (on-slide appendix) and a negative antibody control (buffer, no primary antibody).

## **Kaplan-Meier curve analysis**

The Kaplan-Meier method was used to visualize survival data of OSA patients from the dataset GSE21257 retrieved from the Gene Expression Omnibus database. This dataset includes genome-wide gene expression profiles from the pre-chemotherapy biopsies of OSA patients who developed metastases within 5 years (n=34) and patients who did not develop metastases within 5 years (n=19) (Buddingh *et al*. 2011). Patients were stratified according to *CKB* and *CDH2* expression (upper quartile high and the rest low expression group). The statistical significance of the differences in metastasis-free survival between the patient groups was tested using the log-rank test; the Cox proportional hazard model was used to describe differences in metastasis-free survival between the patient groups by hazard ratios.

**References**

Anderson WJ, Doyle LA. Updates from the 2020 World Health Organization Classification of Soft Tissue and Bone Tumours. Histopathology. 2021;78(5):644-657.

Buddingh EP, Kuijjer ML, Duim RA, Bürger H, Agelopoulos K, Myklebost O, Serra M, Mertens F, Hogendoorn PC, Lankester AC, Cleton-Jansen AM. Tumor-infiltrating macrophages are associated with metastasis suppression in high-grade osteosarcoma: a rationale for treatment with macrophage activating agents. Clin Cancer Res. 2011;17(8):2110-2119.

Miquelestorena-Standley E, Jourdan ML, Collin C, Bouvier C, Larousserie F, Aubert S, Gomez-Brouchet A, Guinebretière JM, Tallegas M, Brulin B, Le Nail LR, Tallet A, Le Loarer F, Massiere J, Galant C, de Pinieux G. Effect of decalcification protocols on immunohistochemistry and molecular analyses of bone samples. Mod Pathol. 2020;33(8):1505-1517.
