# Supplementary figures and images for: Creatine kinase B, a downstream effector of c-Myb, controls migration of osteosarcoma cells via regulation of N-cadherin
Source: Cancer Cell Int. 2025 Dec 5;26:5. doi: 10.1186/s12935-025-04087-0 (PMC12797693; doi:10.1186/s12935-025-04087-0)

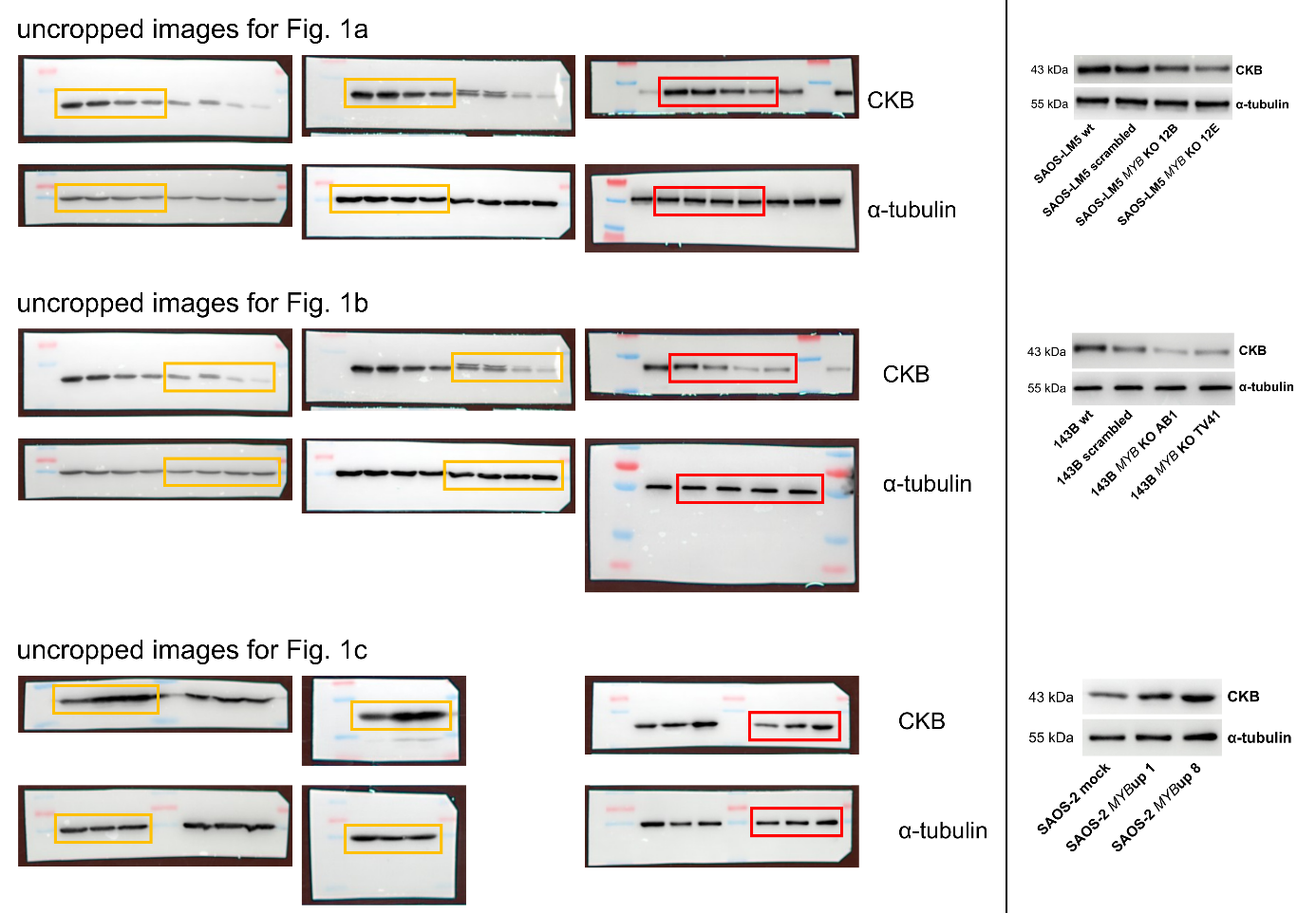


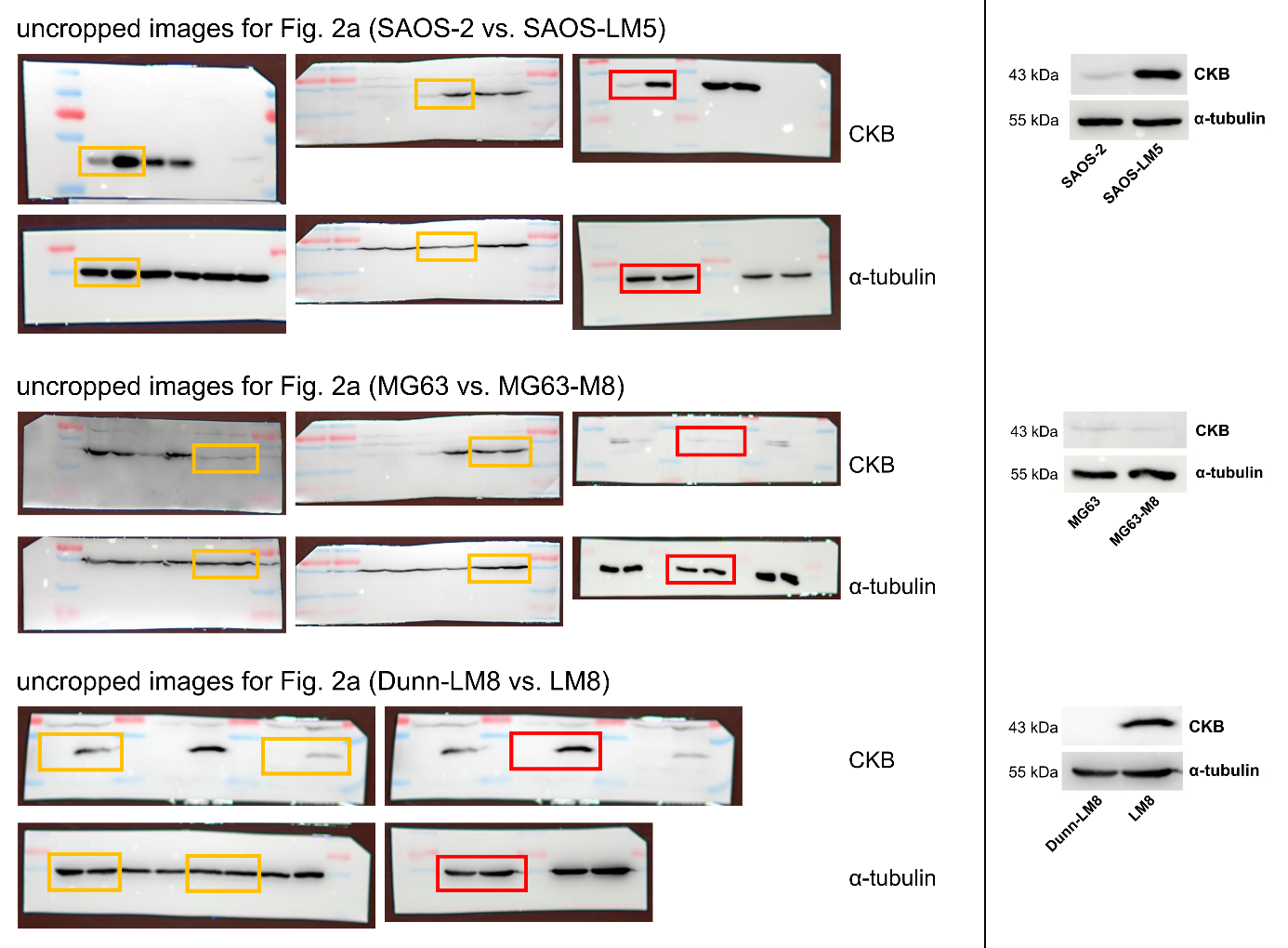


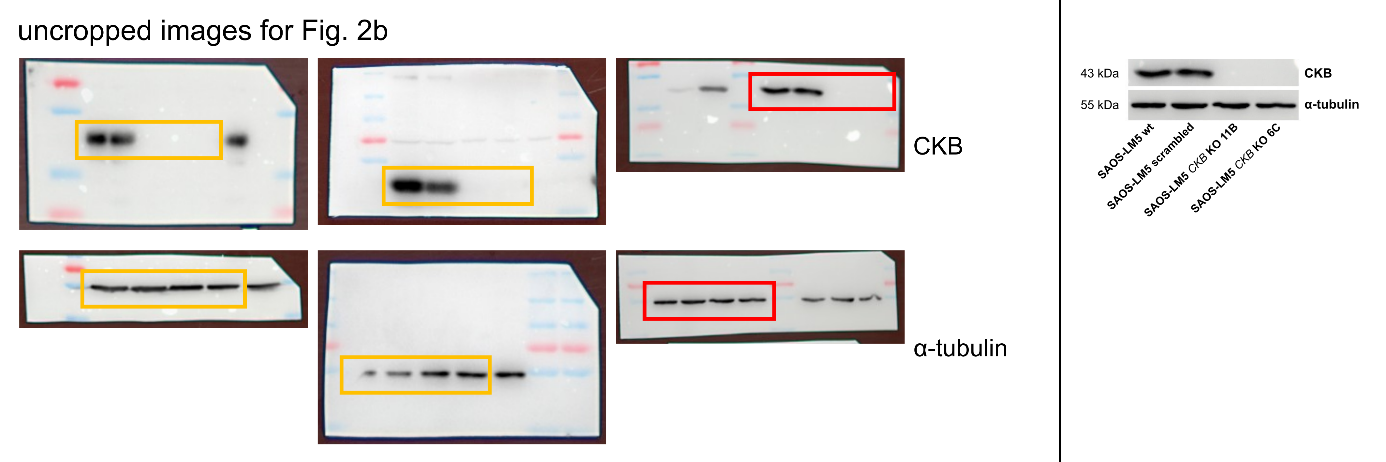


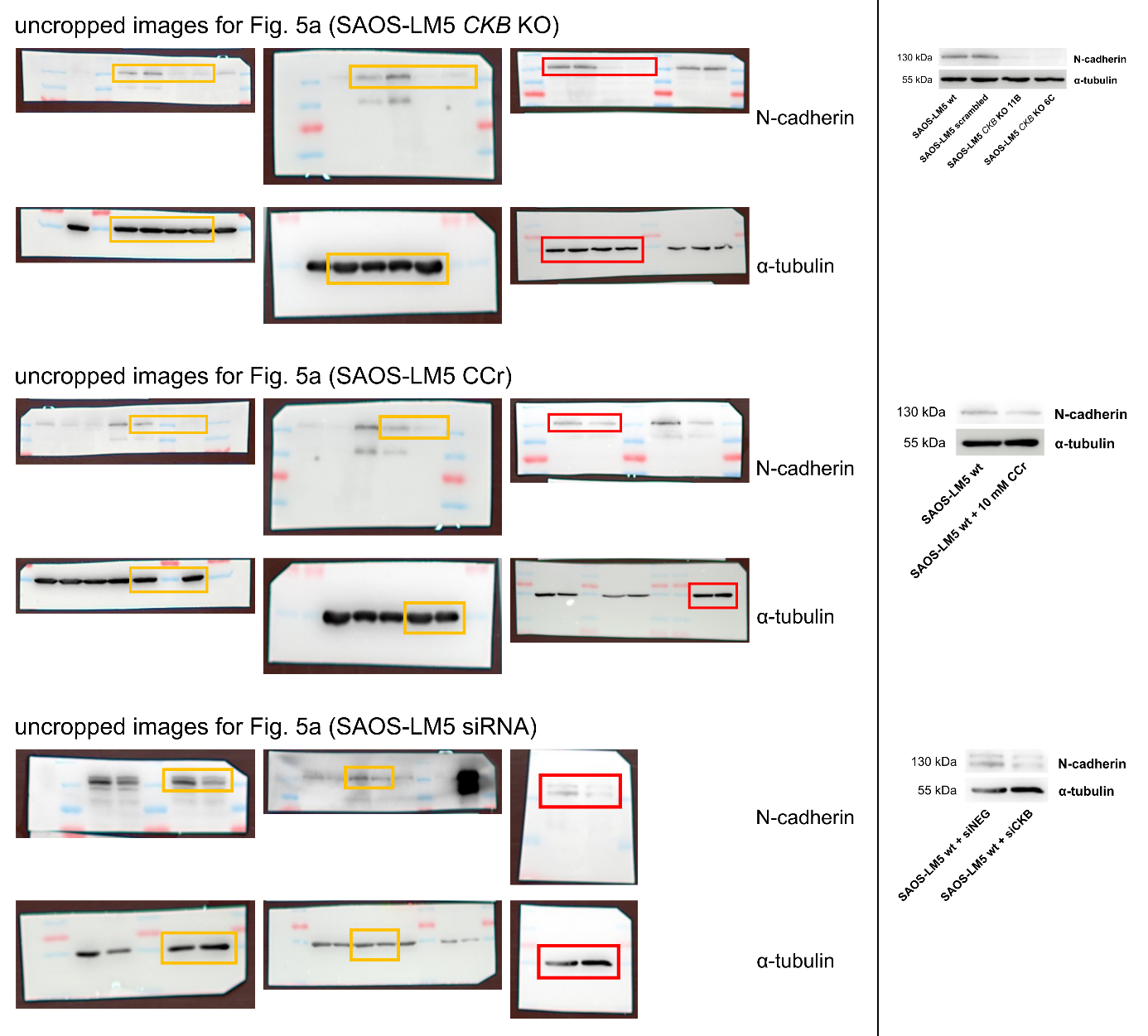


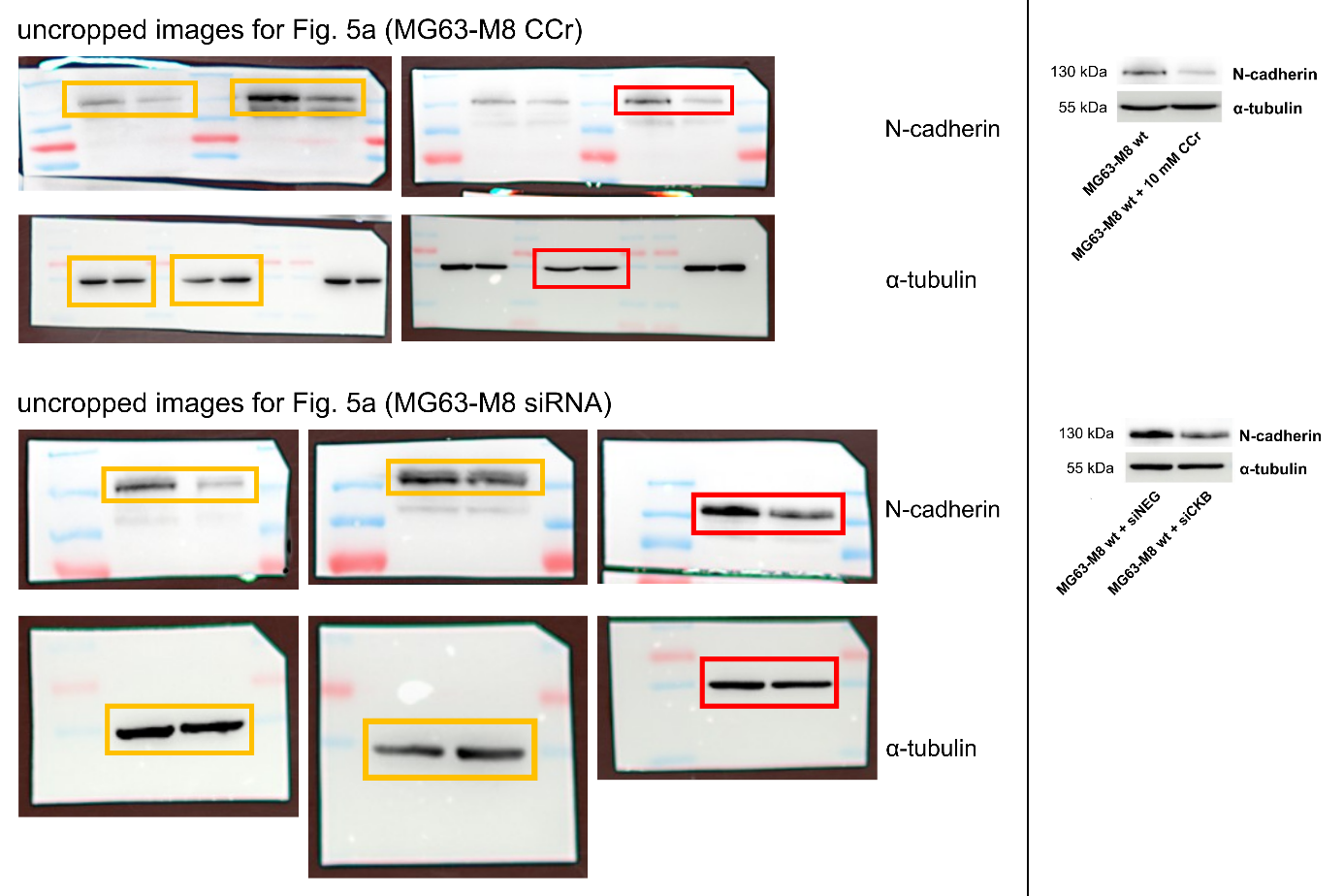


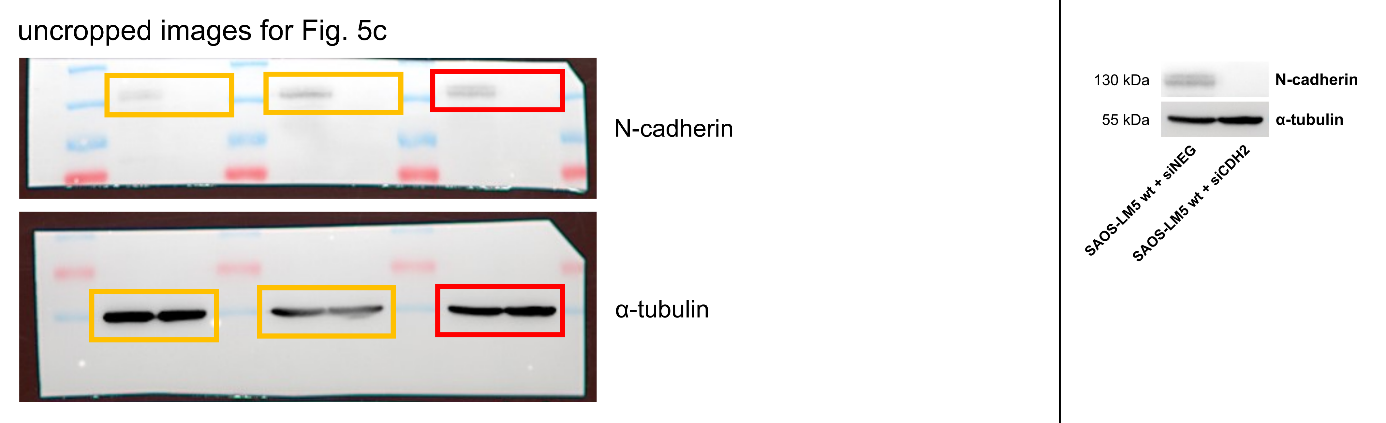


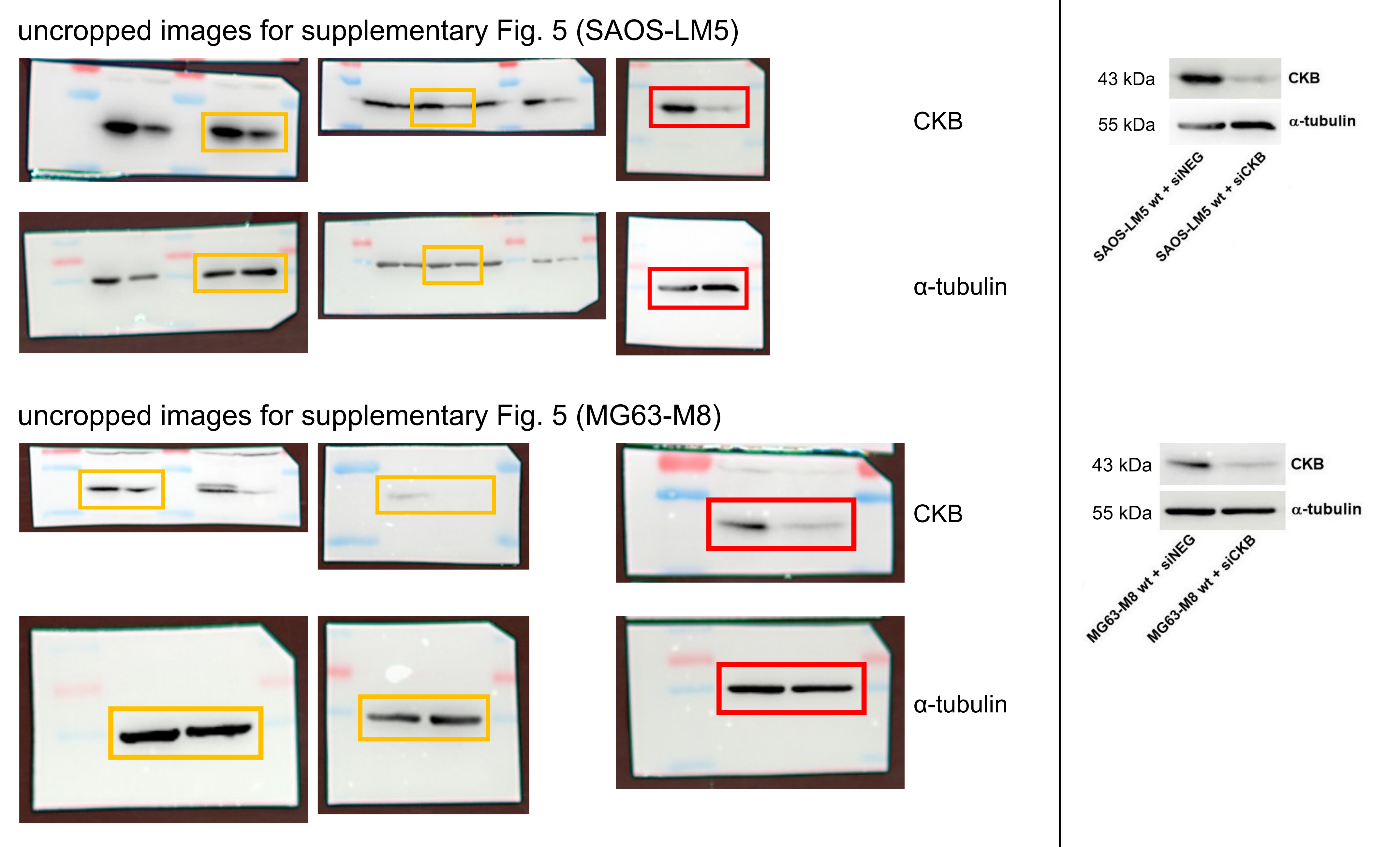


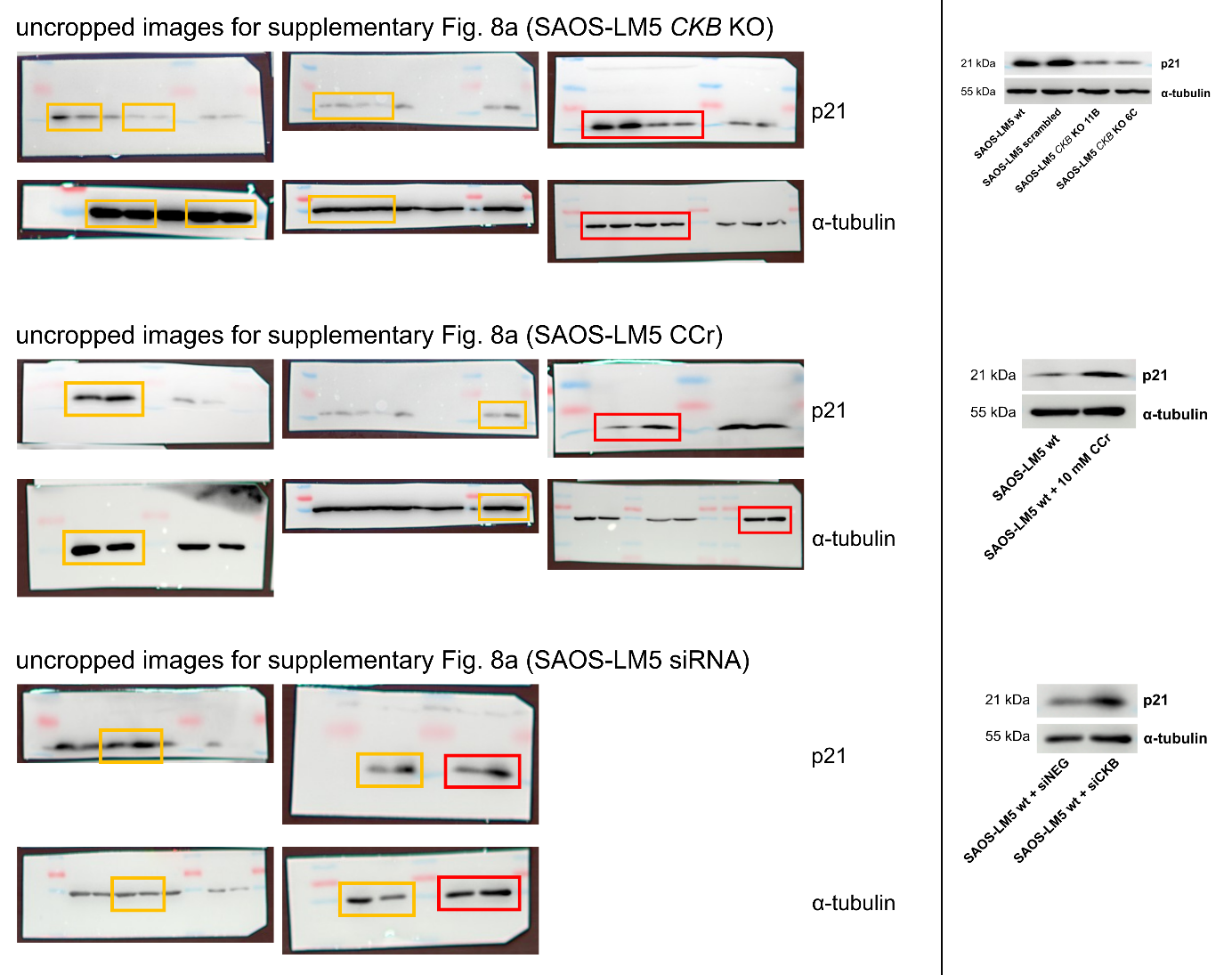


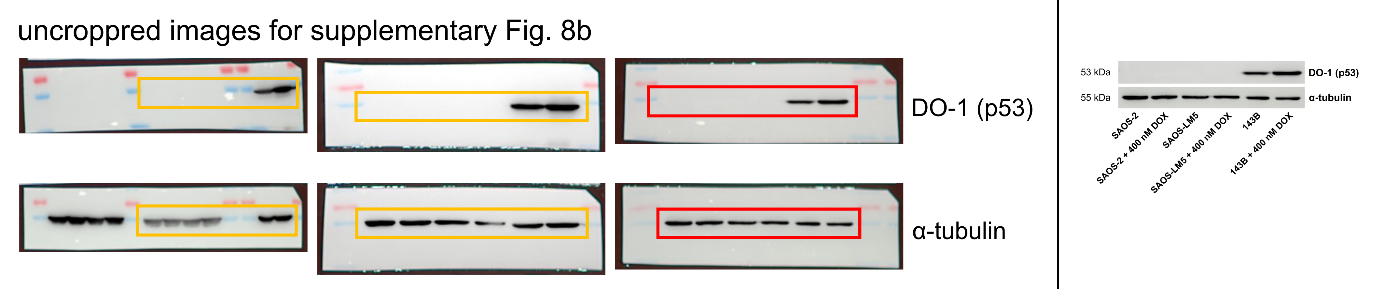

Supplement: Supplementary file 9 — Supplementary Material 9 - uncropped WB [file 12935_2025_4087_MOESM9_ESM.docx]
